# Anticipative information in a Brownian−Poisson market

**DOI:** 10.1007/s10479-022-05060-0

**Published:** 2022-11-27

**Authors:** Bernardo D’Auria, Jose A. Salmeron

**Affiliations:** 1 Department of Mathematics “Tullio Levi-Civita”, University of Padova, Via Trieste, 63, 35131 Padua, Italy; 2 Department of Statistics, University Carlos III of Madrid, Av. de la Universidad, 30, 28911 Leganés, Madrid, Spain

**Keywords:** Optimal portfolio, Malliavin calculus, Clark–Ocone formula, Insider information, Value of the information, 91G10, 60H7, 93E12

## Abstract

The anticipative information refers to some information about future events that may be disclosed in advance. This information may regard, for example, financial assets and their future trends. In our paper, we assume the existence of some anticipative information in a market whose risky asset dynamics evolve according to a Brownian motion and a Poisson process. Using Malliavin calculus and filtration enlargement techniques, we derive the information drift of the mentioned processes and, both in the pure jump case and in the mixed one, we compute the additional expected logarithmic utility. Many examples are shown, where the anticipative information is related to some conditions that the constituent processes or their running maximum may verify, in particular, we show new examples considering Bernoulli random variables.

## Introduction

In this paper we focus on the study of the presence of some anticipative information in a market composed of a bank bond and a risky asset, the latter one driven by a Brownian motion $W=(W_t, \, 0 \le {t} \le {T})$ and a compensated Poisson process ${\tilde{N}} = (N_t-\int _0^t\lambda _s ds,\,0\le t\le T)$ with positive intensity process $\lambda =(\lambda _t, \, 0 \le {t} \le {T})$. In the optimal portfolio problem, a non-informed agent is looking for maximizing her expected logarithmic gains at the end of a trading period $T>0$, while playing with the natural information flow $\mathbb {F}:=\{\mathcal {F}_t\}_{0\le t\le T}$ with $\mathcal {F}_t:=\sigma (W_s,N_s : 0\le s \le t)$. She will be referred to as the $\mathbb {F}$-agent. In addition, we assume that there exists an agent who is informed about a random variable $G\in \mathcal {F}_T$ containing some anticipative information about the path of *W* and/or *N*. The anticipative filtration will be the initial enlargement $\mathbb {G}:= \mathbb {F}\vee \sigma (G)$ and the agent playing with it will be referred to as the $\mathbb {G}$-agent.

Filtration enlargement is a stochastic calculus technique that allows modeling the incorporation of additional non-adapted information. It has multiple applications, including insider trading or, more general, asymmetric information. We refer to the Bibliographic Notes in Chapter VI of Protter (2005) for a detailed overview on this subject. In the seminal paper Amendinger et al. (1998), it is shown that if the dynamics of the risky asset do not include the discontinuous part *N*, then the additional gain under logarithmic utility is given by the entropy of the random variable *G* when it is purely atomic. After that, much progress has been made in the analysis of the additional information in the Brownian case, see Grorud and Pontier (1998) and Amendinger et al. (2003) for the main references. The research on the Poisson process in the initial enlargement framework started with Ankirchner (2008) in which the existence of a compensator is analyzed. Although the entropy is considered, the additional gain of an informed $\mathbb {G}$-agent in the optimal portfolio problem has not been studied. In Di Nunno et al. (2006), a similar framework is studied, however they mainly focused on enlargements that disclose the exact value of the terminal driving processes and their objective is to compute the optimal portfolio without discussing the additional expected logarithmic utility.

Our main motivation is to get an expression for the investor’s additional gains in a market whose risky asset dynamics depend also on a Poisson process, in the same spirit of the analysis done in Theorem 4.1 of Amendinger et al. (1998). This is achieved in Theorem 3.11 for the pure jump case and in Theorem 4.6 for the mixed Brownian–Poisson market. Another novelty of our paper is in the kind of examples that we choose to model the additional information. We consider both $G = \tau \wedge T$, being $\tau$ the time of the first jump of *N*, and $G = \mathbbm {1}_{\{b_1\le N_T\le b_2\}}$, for some constants $b_1,b_2 \in ~\mathbb {N}$. We work also with $G = \mathbbm {1}_{\{a_1\le W_T\le a_2\}\times \{b_1\le N_T\le b_2\}}$, where the $\mathbb {G}$-agent knows if the pair $(W_T,N_T)$ falls within a certain rectangle or not. However, as the main example we consider $G = \mathbbm {1}_{\{a_1\le M_T\le a_2\}\times \{b_1\le J_T\le b_2\}}$ being $M_T:= \sup _{0\le s \le T} W_s$ and $J_T:= \sup _{0\le s \le T}{\tilde{N}}_s$ in which the $\mathbb {G}$-agent knows whether the running maximum processes will be in a certain region or not. Finally, we present an example in which the process $\lambda$ is truly related with *W*, $\lambda _t = 1 +\mathbbm {1}_{\{W_T\ge 0\}}$, $\forall \,t\le T$.

For the majority of our computations, we use Malliavin calculus techniques, we suggest  Di Nunno et al. (2009) or Nualart (2006) for a general overview on this variational calculus. Malliavin Calculus was applied to the optimal portfolio problem in Ocone and Karatzas (1991) via Clark–Ocone formula. With respect to the enlargement of filtration theory, we highlight Imkeller et al. (2001) for a methodology to compute the information drift in initial enlargements and Brownian setting. In particular, some Malliavin regularity assumptions on the conditional densities are assumed in order to drop the so-called *Jacod hypothesis*. In addition, Corcuera et al. (2004) analyzed the case in which the information is becoming more precise via progressive enlargements. Nualart and Vives (1990) applied for the first time the Malliavin approach to the Poisson process while Mensi and Privault (2003) mimicked the Malliavin methodology of Imkeller et al. (2001) for the Poisson process in the context of initial enlargement of filtrations. Finally Wright et al. (2018) weakened some assumptions of the latter paper by assuming the Malliavin derivative were in $L^2(\textrm{d}t\times \textrm{d}\varvec{P})$. Nowadays, the optimal portfolio problem with non-continuous assets is still an active topic of research, for example in Chau et al. (2018), they analyse additional gains generated by an initial enlargement via super-hedging, or Bellalah et al. (2020), that deals with an example related to the Covid-19 crisis. See also Colaneri et al. (2021), where the value of the market price of risk is compared under different information flows.

The paper is organised as follows. In Sect. 2 we describe the framework and we introduce the notation. In Sect. 3 we consider the purely jump market and we get the explicit expression of the compensator of the Poisson process for $\mathcal {F}_T^N$-measurable random variables. The main examples of this section are about the time of the first jump of *N* and the terminal value of the Poisson process, that is, $G=\mathbbm {1}_{\{N_T\in B\}}$ being *B* an interval. In Sect. 3.1, we state Theorem 3.11 in which we get a nice expression for the additional gain of an agent who plays with an initial enlarged filtration. In Sect. 4 we work in a Brownian–Poisson market, in which the additional expected logarithmic utility is computed in Corollary 4.7. The main examples considered in this subsection are $G=\mathbbm {1}_{\{W_T\in A\}\times \{N_T\in B\}}$ and $G=\mathbbm {1}_{\{M_T\in A\}\times \{J_T\in B\}}$ for *A*, *B* some given intervals. In Sect. 5 we construct a model where the Poisson intensity $\lambda$ is an anticipative function of *W*.

## Model and notation

Let $(\Omega , \mathcal {F}_T,\varvec{P},\mathbb {F})$ be a filtered probability space, where the filtration $\mathbb {F}:= \{\mathcal {F}_t\}_{0\le t \le T}$ is assumed to be complete and right-continuous. We assume that the agent is going to invest in a market composed by two assets in a finite horizon time $T>0$. The first one is a risk-less bond $D=(D_t, \, 0 \le {t} \le {T})$ and the second one is a risky stock $S=(S_t, \, 0 \le {t} \le {T})$. The dynamics of both are given by the following SDEs,

1a $$\begin{aligned} \frac{dD_t}{ D_t}&=\rho _tdt,\quad D_0 = 1 \end{aligned}$$

1b $$\begin{aligned} \frac{dS_t}{S_{t-}}&= \mu _tdt + \sigma _tdW_t + \theta _t d\tilde{N}_t ,\quad S_0 = s_0 > 0 , \end{aligned}$$

where $W=(W_t, \, 0 \le {t} \le {T})$ is a Brownian motion and $N = (N_t, \, 0 \le {t} \le {T})$ is a Poisson process with strictly positive intensity $\lambda = (\lambda _t, \, 0 \le {t} \le {T})$, while the compensated version of the Poisson process is defined as $\tilde{N}_t := N_t - \int _0^t \lambda _s ds$ with $\int _0^T \lambda _s ds < +\infty$, $\textrm{d}\varvec{P}\text {-a.s.}$ We assume that *W* and *N* are independent and, to ensure that $S=(S_t, \, 0 \le {t} \le {T})$ is well-defined,

2 $$\begin{aligned} -1<\theta _t\ ,\quad \textrm{d}t\times \textrm{d}\varvec{P}-\text {a.s.} \end{aligned}$$

The measurability of $\lambda$ will be specified in each section. The natural filtration $\mathbb {F}=\{\mathcal {F}_t\}_{0\le t\le T}$ is generated by the Brownian motion and the Poisson process, and it is augmented by the zero $\varvec{P}$-measure sets, $\mathcal {N}$:

$$\begin{aligned} \mathcal {F}_t := \sigma (W_s ,\ N_s:\ 0\le s \le t)\vee \mathcal {N}. \end{aligned}$$

We use the notation $\mathbb {F}^W:=\{\mathcal {F}^W_t\}_{0\le t\le T}$ and $\mathbb {F}^N:=\{\mathcal {F}^N_t\}_{0\le t\le T}$ to refer to the natural filtration of *W* and *N*, respectively. About the market coefficients, in (1a) and (1b) we assume that they are $\mathbb {F}$-predictable processes that satisfy the following integrability condition

3 $$\begin{aligned} \varvec{E}\left[ \int _0^T\left( |\rho _s|+|\mu _s|+\sigma _s^2+\theta _s^2\right) ds\right] <+\infty . \end{aligned}$$

By $\varvec{E}$ and $\varvec{V}$ we refer to the expectation and the variance operators of a given random variable under the measure $\varvec{P}$. Given a $\sigma$-algebra $\mathcal {F}$, by $\varvec{E}[\cdot \vert \mathcal {F}]$ and $\varvec{V}[\cdot \vert \mathcal {F}]$ we denote the conditional expectation and the conditional variance. We introduce the filtration $\mathbb {H}$, that will be the filtration used to define the allowed strategies, and that depending on the agent will coincide with the filtrations $\mathbb {F}$ and $\mathbb {G}$. We define $L^2(\Omega ,\mathcal {H}_T,{\textrm{d}t\times \textrm{d}\varvec{P}},\mathbb {H})$, or simply $L^2({\textrm{d}t\times \textrm{d}\varvec{P}})$ when the reference filtration is clear, as the space of all $\mathbb {H}$-adapted processes *X* such that $\int _0^T \varvec{E}\left[ X_s^2\right] ds <+ \infty$.

Using the previous set-up, it is assumed that an agent can control her portfolio by a *self-financing* process $\pi =(\pi _t, \, 0 \le {t} \le {T})$, with the aim to maximize her expected logarithmic gains at the finite horizon time. It represents the fraction of the monetary unit amount invested in the risky asset. We denote by $X^\pi =(X^\pi _t, \, 0 \le {t} \le {T})$ a positive process to model the wealth of the portfolio of the investor under the strategy $\pi$. The dynamics of the wealth process are given by the following SDE, for $0 \le t \le T$,

4 $$\begin{aligned} \frac{dX_t^{\pi }}{X_{t-}^{\pi }} = (1-\pi _t) \frac{dD_t}{D_t} + \pi _t \frac{dS_t}{S_{t-}} ,\quad X^{\pi }_0=x_0 > 0, \end{aligned}$$

and by using the evolution of both assets given in (1) we get

$$\begin{aligned} \frac{dX^{\pi }_t}{X^{\pi }_{t-}}&= (1-\pi _t) \rho _tdt + \pi _t \left( \mu _tdt + \sigma _t dW_t+\theta _t d{\tilde{N}}_t \right) ,\quad X^{\pi }_0=x_0, \end{aligned}$$

where the SDE is well-defined on the probability space $(\Omega , \mathcal {F}_T,\varvec{P},\mathbb {F})$. Before giving a proper definition of the set of processes $\pi$ that we consider, we look for the natural conditions they should satisfy. Applying the Itô formula to the dynamics of the risky asset given by (1b), we get an explicit solution as follows,

$$\begin{aligned} \ln \frac{S_t}{s_0} = \int _0^t \left( \mu _s -\frac{1}{2}\sigma ^2_s+ \lambda _s(\ln (1+\theta _s) -\theta _s)\right) ds + \int _0^t \sigma _sdW_s + \int _0^t\ln (1+\theta _s)d{\tilde{N}}_s. \end{aligned}$$

If we apply the Itô formula to the wealth process we get,

5 $$\begin{aligned} \ln \frac{X^{\pi }_t}{x_0} = \int _0^{t}\left( \rho _s + \pi _s (\mu _s-\rho _s) - \frac{1}{2}\pi ^2_s\sigma ^2_s +\lambda _s(\ln (1+\pi _s\theta _s)-\pi _s\theta _s)\right) ds \end{aligned}$$

provided that these integrals are well-defined. To ensure this, we assume the following integrability condition,

6 $$\begin{aligned} \varvec{E}\left[ \int _0^T\left( |\pi _s||\mu _s-\rho _s|+\pi ^2_s\sigma ^2_s + \pi ^2_s\theta ^2_s\right) ds \right] < +\infty . \end{aligned}$$

In order to guarantee that $X^{\pi }$ is well-defined, we impose that

7 $$\begin{aligned} 1+\pi _t\theta _t> 0,\quad {\textrm{d}t\times \textrm{d}\varvec{P}-\text {a.s.}} \end{aligned}$$

Now, we can properly define the optimization problem as the supremum of the expected logarithmic gains of the agent’s wealth at the finite horizon time *T*.

8 $$\begin{aligned} \mathbb {V}_T^{\mathbb {H}}:= \sup _{\pi \in \mathcal {A}(\mathbb {H})} \varvec{E}\left[ \ln X^{\pi }_T\vert X^\pi _0 = x_0\right] ,\quad \mathbb {H}\supseteq \mathbb {F}. \end{aligned}$$

Finally we give the definition of the set $\mathcal {A}(\mathbb {H})$ made of all admissible strategies for the $\mathbb {H}$-agent, that is the one playing with information flow $\mathbb {H}\supseteq \mathbb {F}$.

### Definition 2.1

In the financial market (1a)–(1b), we define the set of admissible strategies $\mathcal {A}(\mathbb {H})$ as the set of portfolio processes $\pi$ predictable with respect to the filtration $\mathbb {H}$ which satisfy the conditions (6) and (7).

### Definition 2.2

The additional expected logarithmic utility of a filtration $\mathbb {H}~\supseteq ~\mathbb {F}$ is given by

$$\begin{aligned} \Delta \mathbb {V}_T^\mathbb {H}= \mathbb {V}_T^{\mathbb {H}} - \mathbb {V}_T^{\mathbb {F}}, \end{aligned}$$

where the quantities on the right-hand side are defined in (8).

In the following statement we summarize the results about the optimal portfolios in markets with Brownian noise, Poisson noise or both. It can be found in Corollary 17 of Di Nunno et al. (2006).

### Proposition 2.3

The optimal strategy $\pi ^*$ for the problem (8) with the information flow $\mathbb {F}$ satisfies

9 $$\begin{aligned} { \mu _t-\rho _t-\sigma _t^2\pi ^*_t - \lambda \frac{\theta _t^2\pi ^*_t}{1+\pi ^*_t\theta _t} = 0.} \end{aligned}$$

In particular, if we have that $\sigma _t > 0$ and $\theta _t=0$ ${\textrm{d}t\times \textrm{d}\varvec{P}}$-a.s., then we recover the classic Merton problem with the optimal strategy satisfying the relation

10 $$\begin{aligned} \pi _t^* = \frac{\mu _t-\rho _t}{\sigma _t^2}. \end{aligned}$$

Finally, if we have that $\sigma _t = 0$, $\theta _t\ne 0$ and ${\textrm{d}t\times \textrm{d}\varvec{P}}$-a.s., then the optimal strategy is given by

11 $$\begin{aligned} \pi _t^* = \frac{\mu _t-\rho _t}{\lambda \theta _t^2-\theta _t(\mu _t-\rho _t)}. \end{aligned}$$

Let $G\in \mathcal {F}_T$ be a real valued random variable modeling some additional information. We introduce the filtration $\mathbb {G}:=\{\mathcal {G}_t\}_{0\le t\le T}$ under which the privileged information is accessible since the beginning time $t=0$, that is

12 $$\begin{aligned} \mathcal {G}_t = \bigcap _{s>t}\left( \mathcal {F}_s \vee \sigma (G) \right) , \end{aligned}$$

We denote with $\varvec{P}^G$ the distribution of *G*, i.e., $\varvec{P}^G(\cdot ) = \varvec{P}(G\in \cdot )$ on $\sigma (G)$ and by $\varvec{P}^G(\cdot \vert \mathcal {F}) = \varvec{P}(G\in \cdot \vert \mathcal {F})$ the corresponding conditional probability with respect to a given $\sigma$-algebra $\mathcal {F}$. The crucial point is to ensure that any $\mathbb {F}$-semimartingale is also a $\mathbb {G}$-semimartingale, that is known in the literature as the $(\mathcal {H}')$ *hypothesis*, see Jeulin and Yor (1978). An approach widely used in the literature to achieve this is known as the *Jacod hypothesis* and assumes that the conditional distributions $\varvec{P}^G(\cdot \vert \mathcal {F}_t)$ is absolutely continuous with respect to $\varvec{P}^G$ for $t\in [0,T)$ a.s. In Theorem 2.5 of Jacod (1985) it is proven that the Jacod hypothesis implies the ($\mathcal {H}$’) hypothesis. However, this assumption sometimes results too restrictive. Therefore, we use the following proposition valid in the framework of Poisson process to compute the information drift under weaker assumptions. We refer to Proposition 3 in Mensi and Privault (2003) for a proof, for a similar result in the Brownian motion framework see Theorem 2.1 in Imkeller et al. (2001). We present a slightly different statement adapted to finite deterministic time *T*.

### Proposition 2.4

Let $G\in \mathcal {F}_T^N$, consider $\mathbb {G}=\mathbb {F}^N \vee \sigma (G)$ and $P_t(\omega , dg):=\varvec{P}\left( G\in dg\vert \mathcal {F}^N_t\right) (\omega )$, with $0\le t \le T$, denote a version of the conditional law of *G* given $\mathcal {F}^N_t$ and assume it has the following representation $P_t(\cdot , dg)=P_0(\cdot , dg)+\int _0^t \phi _s(\cdot , dg) d\tilde{N}_s\ ,\quad 0\le t \le T\ ,$ and there exists a measurable *h* such that $\phi _s(\cdot , dg)=h_s(\cdot , g) P_s(\cdot , dg),$ $0\le s < T$, then $\tilde{N}_\cdot -\int _0^\cdot h_s(\cdot , G)ds$ is a $\mathbb {G}$-local martingale, with $\mathbb {G}=\mathbb {F}^N \vee \sigma (G)$.

The process $h(\cdot , G)$ is usually called the *information drift* and it plays a crucial role in our computations.

To conclude this section, we state the Clark–Ocone formula valid for the case the intensity $\lambda$ is a deterministic process. The operators $D_t$ and $D_{t,1}$ refer to the Malliavin derivative in the Brownian and Poisson cases respectively and we consider their generalizations to $L^2(\varvec{P})$. We refer to Theorem 13.28 in Di Nunno et al. (2009) for the details and a general background.

### Proposition 2.5

Let $\lambda$ be a deterministic process and $G\in L^2(\varvec{P})$ be an $\mathcal {F}_T$-measurable random variable, then the following representation holds,

13 $$\begin{aligned} G = \varvec{E}[G] + \int _0^T \varvec{E}[D_t G\vert \mathcal {F}_t] dW_t + \int _0^T \varvec{E}[D_{t,1} G\vert \mathcal {F}_t] d{\tilde{N}}_t. \end{aligned}$$

## Initial enlargements in a pure Poisson market

In this section, we deal with a market in which the source of the noise of the risky asset is only the Poisson process, i.e., we assume that $\sigma _t = 0$, ${\textrm{d}t\times \textrm{d}\varvec{P}}$-a.s. and the intensity process $\lambda$ is deterministic. Using the predictable representation property (PRP) enjoyed by the compensated Poisson process, we know that for every $\mathcal {F}^N_T$-measurable real valued random variable $G\in L^2(\varvec{P})$, there exists an $\mathbb {F}^N$-predictable process $\varphi \in L^2({\textrm{d}t\times \textrm{d}\varvec{P}})$ such that

14 $$\begin{aligned} G = \varvec{E}[G] + \int _0^T \varphi _s d{\tilde{N}}_s, \end{aligned}$$

usually called the *non-anticipative derivative* of *G*, see Di Nunno (2007). Let $B\in \mathcal {B}(\mathbb {R})$ be a subset in the Borel $\sigma$-algebra on $\mathbb {R}$ and consider the following PRP,

15 $$\begin{aligned} \mathbbm {1}_{\{G\in B\}} = \varvec{P}^G(B) + \int _0^T \varphi _s(B)d{\tilde{N}}_s, \end{aligned}$$

where by the predictable process $\varphi (B)=(\varphi _t(B):0\le t\le T)$ we denote the unique one within the Hilbert space $L^2({\textrm{d}t\times \textrm{d}\varvec{P}})$ that satisfies (15) for a fixed *B*.

In the next lemma we prove that $\varphi (\cdot )$ is a vector measure, we refer to Diestel and Uhl (1977) for a general background on the vector measure theory. We make the following assumption, for more details we refer to Definition 4 of Diestel and Uhl (1977).

### Assumption 3.1

The vector measure $\varphi$ is of bounded variation.

### Remark 3.2

Assumption 3.1 is trivially satisfied for a bounded discrete random variable as the variation of the vector measure $\varphi$ is bounded above by a constant times the number of values assumed by the random variable.

In the following lemma we state the Radon-Nikodym derivative for the Hilbert valued random measure $\varphi$.

### Lemma 3.3

The set function $B\longrightarrow \varphi (B)$, with $B\in \mathcal {B}(\mathbb {R})$, is a countably additive $L^2(\textrm{d}t\times \textrm{d}\varvec{P})$-valued vector measure and there exists a set of processes $\psi = (\psi ^g,\, g\in {{\,\textrm{Supp}\,}}(G))$ such that $\psi ^g = (\psi ^g_t, \, 0 \le {t} \le {T})\in L^1(\varvec{P}^G)$ satisfying

$$\begin{aligned} \varphi _t(B) = \int _B \psi _t^g\varvec{P}^G(dg),\quad B\subset \mathbb {R}. \end{aligned}$$

### Proof

Let $\{B_i\}_{i=1}^\infty \subset \mathcal {B}(\mathbb {R})$ be a disjoint sequence of subsets satisfying $B = \cup _{i=1}^\infty B_i.$ Then $\mathbbm {1}_{\{G\in B\}} = \sum _{i=1}^\infty \mathbbm {1}_{\{G\in B_i\}}$ $\varvec{P}$-a.s. in $L^2(\textrm{d}\varvec{P})$ (see Example 3 in Diestel and Uhl (1977)). Then, using the PRP we get

$$\begin{aligned} \mathbbm {1}_{\{G\in B\}} = \sum _{i=1}^\infty \mathbbm {1}_{\{G\in B_i\}} = \varvec{P}(G\in B) + \sum _{i=1}^\infty \int _0^T \varphi _t(B_i)d{\tilde{N}}_t = \int _0^T \sum _{i=1}^\infty \varphi _t(B_i)d{\tilde{N}}_t , \end{aligned}$$

In the second equality we applied the $\sigma$-additivity of the probability measure $\varvec{P}$ while in the third one, thanks to $\varphi (B)\in L^2(\textrm{d}t\times \textrm{d}\varvec{P})$ for any $B\in \mathcal {B}(\mathbb {R})$, we used the stochastic Fubini theorem, see Lemma A.1.1 in Mandrekar and Rüdiger (2015). By the uniqueness of the representation we deduce that $\varphi (B) = \sum _{i=1}^\infty \varphi (B_i)$ and we conclude that $\varphi$ is a $L^2(\textrm{d}t\times \textrm{d}\varvec{P})$-vector measure. As $\varphi \ll \varvec{P}^G$ on $\sigma (G)$, the last claim of the lemma follows by Proposition 2.1 of Kakihara (2011). $\square$

Therefore, Lemma 3.3 guarantees that there exists a set of processes $\psi ^g = (\psi ^g_t, \, 0 \le {t} \le {T})$ with $g\in {{\,\textrm{Supp}\,}}(G)$ such that the PRP given in (15) can be written as follows

16 $$\begin{aligned} \mathbbm {1}_{\{G\in B\}} = \varvec{P}^G(B) + \int _0^T \int _B \psi _s^g\varvec{P}^G(dg)d{\tilde{N}}_s. \end{aligned}$$

In particular, by assuming measurability on $\psi$ in all variables, the following representation

17 $$\begin{aligned} \mathbbm {1}_{\{G\in dg\}} = \varvec{P}^G(dg) + \int _0^T \psi _s^g d{\tilde{N}}_s\, \varvec{P}^G(dg) \end{aligned}$$

holds true, where Fubini’s theorem has been applied. When *G* is purely atomic, the PRP (17) reduces to

18 $$\begin{aligned} \mathbbm {1}_{\{G = g\}} = \varvec{P}(G = g) + \int _0^T \psi _s^g d{\tilde{N}}_s \,\varvec{P}(G = g). \end{aligned}$$

The following result provides the information drift in terms of the process $\psi$.

### Lemma 3.4

Let $G\in L^2(\varvec{P})$ be an $\mathcal {F}_T^N$-measurable random variable satisfying Assumption 3.1, then the process $\gamma ^G=(\gamma ^G_t, \, 0 \le {t} <{T})$ defined as

19 $$\begin{aligned} \gamma _t^g := \frac{\psi ^g_t\varvec{P}^G(dg)}{\varvec{P}^G(dg\vert \mathcal {F}^N_t)}\ ,\quad g\in \text {Supp}(G) \end{aligned}$$

satisfies that ${\tilde{N}}_\cdot -\int _0^\cdot \lambda _s\gamma _s^G ds$ is a $\mathbb {G}$-local martingale, provided it is well-defined. $\gamma ^G$ is referred to as the information drift.

### Proof

Once we got (16), we proceed similarly to Proposition 2.4 but in the case of $\lambda$ deterministic. Let $A\in \mathcal {F}_s$ and $B\in \sigma (G)$, then

$$\begin{aligned}&\varvec{E}[\mathbbm {1}_A\mathbbm {1}_{\{G\in B\}}({\tilde{N}}_t-{\tilde{N}}_s)] = \varvec{E}\left[ \mathbbm {1}_A\left( \varvec{P}^G(B)+ \int _0^T \int _B \psi ^{g}_s \varvec{P}^G(dg) d{\tilde{N}}_s\right) ({\tilde{N}}_t-{\tilde{N}}_s)\right] \\&\quad = \varvec{E}\left[ \mathbbm {1}_A \int _s^t\int _B \psi ^g_u \varvec{P}^G(dg) dN_u \right] = \varvec{E}\left[ \mathbbm {1}_A \int _s^t\int _B \lambda _u\gamma _u^g\varvec{P}^G(dg\vert \mathcal {F}_u) du \right] \\&= \varvec{E}\left[ \mathbbm {1}_A \int _s^t \varvec{E}[\lambda _u\gamma _u^G \mathbbm {1}_{\{G\in B\}}\vert \mathcal {F}_u] du \right] = \varvec{E}\left[ \mathbbm {1}_A\mathbbm {1}_{\{G\in B\}} \int _s^t\lambda _u\gamma _u^Gdu \right] \ . \end{aligned}$$

As the computation holds true for any $A\in \mathcal {F}_s,\,B\in \sigma (G)$ we conclude that

$$\begin{aligned} \varvec{E}[{\tilde{N}}_t-{\tilde{N}}_s\vert \mathcal {G}_s] = \varvec{E}\left[ \int _s^t\lambda _u\gamma _u^Gdu\vert \mathcal {G}_s\right] \end{aligned}$$

and the result holds true. $\square$

In order to assure that the optimal strategy in $\mathbb {G}$ is well defined, in the sense of equation (7), we are going to assume some conditions on the information drift $\gamma ^G$. In particular,

20 $$\begin{aligned} \gamma _s^G>-1 ,\quad \textrm{d}t\times \textrm{d}\varvec{P}-a.s. \end{aligned}$$

A condition similar to (20) appears in Section 4.1 of Grorud (2000) where they study Poisson processes in enlarged filtrations.

### Corollary 3.5

If $${{\,\textrm{Supp}\,}}(G)=\{0,1,\ldots ,n\}$$, then

$$\begin{aligned} \gamma _t^g = \varphi ^g_t \frac{\mathbbm {1}_{\{G=g\}} -\varvec{E}[\mathbbm {1}_{\{G=g\}}\vert \mathcal {F}_t]}{\varvec{V}[\mathbbm {1}_{\{G=g\}}\vert \mathcal {F}_t]}\ ,\quad \varphi ^g_t = \psi ^g_t\varvec{P}(G=g),\quad g\in {{\,\textrm{Supp}\,}}(G). \end{aligned}$$

In addition, if ${{\,\textrm{Supp}\,}}(G)=\{0,1\}$, then

21 $$\begin{aligned} \gamma _t^G = (-1)^{G+1}\varphi _t\frac{G-\varvec{E}[G\vert \mathcal {F}^N_t]}{\varvec{V}[G\vert \mathcal {F}^N_t]}. \end{aligned}$$

### Proof

The former statement comes directly from (19) while for the latter we use the fact that $G = \mathbbm {1}_{\{G=1\}}$ and, by the uniqueness of the representation, we conclude that $\varphi = \varphi ^1$ and $\varphi ^1 = -\varphi ^0$, where by $\varphi ^0$ and $\varphi ^1$ we refer to the non-anticipative derivative of $\mathbbm {1}_{\{G=0\}}$ and $\mathbbm {1}_{\{G=1\}}$ respectively. Using that

$$\begin{aligned} \varvec{E}[G\vert \mathcal {F}^N_t] = \varvec{P}(G=1\vert \mathcal {F}^N_t),\quad \varvec{V}[G\vert \mathcal {F}^N_t] = \varvec{P}(G=1\vert \mathcal {F}^N_t)(1-\varvec{P}(G=1\vert \mathcal {F}^N_t)), \end{aligned}$$

the result follows by applying (14) and (18). $\square$

### Remark 3.6

Note that if ${{\,\textrm{Supp}\,}}(G)=\{0,1\}$ we can express

22 $$\begin{aligned} \gamma _t^g= \frac{{(-1)^g}\,\varphi _t}{\varvec{P}(G = 0\vert \mathcal {F}^N_t)-g} \ ,\quad g\in \{0,1\}, \end{aligned}$$

where $\varphi$ is the non-anticipative derivative of the Bernoulli random variable *G*.

By using the Clark–Ocone formula we can deduce that $\varphi _t = \varvec{E}[D_{t,1} G\vert \mathcal {F}^N_t]$ $\textrm{d}t\times \textrm{d}\varvec{P}-a.s.$, which allows to compute some interesting examples. Following Solé et al. (2007), we introduce the following operator

23 $$\begin{aligned} \Psi _{t,1} G := {G(\omega _{(t,1)})-G(\omega )}, \end{aligned}$$

being $\omega _{(t,1)}$ the modification of the trajectory $\omega$ by adding a new jump of size 1 at time *t*. In Proposition 5.4 of Solé et al. (2007) it is proved that if $\Psi _{t,1} G\in L^2({\textrm{d}t\times \textrm{d}\varvec{P}})$, then this operator coincides with the usual Malliavin derivative, in the case of the Poisson process we have that $\Psi _{t,1} G = D_{t,1} G$ $\textrm{d}t\times \textrm{d}\varvec{P}-a.s.$

In the next example, we apply Lemma 3.4 to an enlargement with a continuous random variable. In particular, we consider that the informed agent knows when the Poisson process jumps for the first time. Ernst and Rogers (2020, Problem 2) solved a similar problem via the HJB equation.

### Example 3.7

We consider the random time $\tau =\inf \{t{\ge 0}:N_t=1\}$ which represents the time of the first jump of the point process *N* and we define $G=\tau \wedge T$. In this example, we study the enlargement of filtration $\mathbb {G}\supset \mathbb {F}$ induced by *G* and we compute the information drift given by Lemma 3.4 via the PRP (17). We fix $g\in [0,T]$ and consider the following Clark–Ocone formula

$$\begin{aligned} \mathbbm {1}_{\{G\le g\}} = \varvec{P}(G\in dg) + \int _0^T\varvec{E}[D_{t,1}\mathbbm {1}_{\{G\le g\}}\vert \mathcal {F}_t]d{\tilde{N}}_t. \end{aligned}$$

We compute the integrand $\varvec{E}[D_{t,1}\mathbbm {1}_{\{G\le g\}}\vert \mathcal {F}_t]$ by using the operator $\Psi$ for any $(t,g)~\in ~[0,T]^2$, defined in (23). Note that, when $g<t$, the perturbation is not modifying the indicator so $\Psi _{t,1}\mathbbm {1}_{\{G\le g\}} = 0$. When $g\ge t$, only on the set $\{\tau (\omega )>g\}$ the operator is not null. In general, it holds the following equation

$$\begin{aligned} \Psi _{t,1} \mathbbm {1}_{\{G\le g\}} = \mathbbm {1}_{\{t\le g\}}\mathbbm {1}_{\{\tau >g\}}. \end{aligned}$$

Since the Clark–Ocone formula is linear, a limiting argument implies that

$$\begin{aligned} D_{t,1}\mathbbm {1}_{\{G\in dg\}} = -\mathbbm {1}_{\{t<g\}}\mathbbm {1}_{\{\tau \in dg \}}+\mathbbm {1}_{\{t\in dg \}}\mathbbm {1}_{\{\tau >g\}}. \end{aligned}$$

By (19), the information drift is

24 $$\begin{aligned} \gamma ^g_t = -\mathbbm {1}_{\{t<g\}}\frac{\varvec{P}(\tau \in dg\vert \mathcal {F}_t^N)}{\varvec{P}(G\in dg\vert \mathcal {F}_t^N)} + \mathbbm {1}_{\{t\in dg\}}\frac{\varvec{P}(\tau > g \vert \mathcal {F}_t^N)}{\varvec{P}(G\in dg\vert \mathcal {F}_t^N)} \ ,\quad g\in [0,T], \end{aligned}$$

where the first component considers the additional information before the jump and second one the information at the time of the jump. Note that, by the first term appearing in (24), we conclude that $\gamma _t^G$ does not satisfy assumption (20) as with positive probability $\gamma _t^G=-1$. This is a clear evidence that there is a strong arbitrage in this enlargement. In general, this is an indication that the anticipative information may generate arbitrage opportunities.

### Example 3.8

Let $G = \mathbbm {1}_{\{ N_T \in B \}}$ with $B=[b_1,b_2]$, and $b_1,b_2\in \mathbb {N}$, $b_2>b_1>0$. We consider the initial enlargement $\mathbb {G}\supset \mathbb {F}$. We compute the process $\varphi$ as follows, $\Psi _{t,1} \mathbbm {1}_{\{N_T \in B \}} = \mathbbm {1}_{\{ N_T+1 \in B\}} - \mathbbm {1}_{\{ N_T \in B\}}$, which obviously satisfies the integrability condition and therefore

$$\begin{aligned} D_{t,1} \mathbbm {1}_{\{ N_T \in B\}} = \mathbbm {1}_{\{ N_T=b_1-1\}} - \mathbbm {1}_{\{ N_T = b_2\}}, \end{aligned}$$

and computing the conditional expectation we obtain the Clark–Ocone formula

$$\begin{aligned} \varphi _t=\varvec{E}\left[ D_{t,1} \mathbbm {1}_{\{ N_T \in B\}} \vert \mathcal {F}_t^N\right]&= \varvec{P}\left( N_T = b_1-1\vert \mathcal {F}^N_t \right) - \varvec{P}\left( N_T = b_2\vert \mathcal {F}_t^N \right) \end{aligned}$$

and the following PRP holds,

25 $$\begin{aligned} \mathbbm {1}_{\{N_T\in B \}} = \varvec{P}(N_T\in B) - \int _0^T \left( \varvec{P}\left( N_T = b_2\vert \mathcal {F}_t^N \right) - \varvec{P}\left( N_T = b_1-1 \vert \mathcal {F}^N_t \right) \right) d{\tilde{N}}_t, \end{aligned}$$

giving the following formula for compensator

26 $$\begin{aligned} \gamma ^G_t ={(-1)^G} \frac{ \varvec{P}\left( N_T = b_1-1\vert \mathcal {F}_t^N \right) - \varvec{P}\left( N_T = b_2 \vert \mathcal {F}^N_t \right) }{\varvec{P}(N_T\in B^{c}\vert \mathcal {F}_t^N)-G}. \end{aligned}$$

A direct computation shows that $\gamma ^1_t >-1$, while for the case of $G=0$, some simulations are applied. In the particular case of $\lambda =T=1$, the computation is direct.

As $\lambda$ is deterministic, we can compute the probabilities as follows

$$\begin{aligned} \varvec{P}(N_t-N_s = n \vert \mathcal {F}^N_s) = e^{-\Lambda (s,t)}\frac{(\Lambda (s,t))^n}{n!} ,\quad \Lambda (s,t):= \int _s^t \lambda _u du,\quad n\in \mathbb {N}, \end{aligned}$$

and the PRP simplifies to

$$\begin{aligned} \mathbbm {1}_{\{N_T\in B \}}&= \varvec{P}(N_T\in B)- \int _0^T e^{-\Lambda (t,T)}\left( \frac{(\Lambda (t,T))^{b_2-N_t}}{(b_2-N_t)!}\mathbbm {1}_{\{N_t\le b_2\}}\right. \\&\quad \left. - \frac{(\Lambda (t,T))^{b_1-N_t-1}}{(b_1-N_t-1)!}\mathbbm {1}_{\{N_t< b_1\}} \right) d{\tilde{N}}_t \end{aligned}$$

and the information drift is

$$\begin{aligned} \gamma ^G_t = {(-1)^G} e^{-\Lambda (t,T)} \frac{ \frac{(\Lambda (t,T))^{b_1-N_t-1}}{(b_1-N_t-1)!}\mathbbm {1}_{\{N_t < b_1\}} - \frac{(\Lambda (t,T))^{b_2-N_t}}{(b_2-N_t)!}\mathbbm {1}_{\{N_t\le b_2\}} }{\varvec{P}(N_T\in B^{c}\vert \mathcal {F}_t^N)-G}. \end{aligned}$$

Note that, in the simplest case of time-homogeneous Poisson process with constant intensity $\lambda > 0$, we have $\Lambda (t,T) = \lambda (T-t)$.

### Additional expected logarithmic utility

Throughout this subsection, to assure that $\mathbb {V}^{\mathbb {F}}_T<\infty$, we assume that the market coefficients satisfy the following relation

27 $$\begin{aligned} 0 < \lambda _t - \frac{\mu _t-\rho _t}{\theta _t} ,\quad \textrm{d}t\times \textrm{d}\varvec{P}-\text {a.s.} \end{aligned}$$

Working in the filtration $\mathbb {F}$, if we take expectation in (5), then

$$\begin{aligned} \varvec{E}\left[ \ln \frac{X^\pi _T}{x_0}\right] = \varvec{E}\left[ \int _0^T \rho _s + \pi _s(\mu _s-\rho _s) + \lambda _s(\ln (1+\pi _s\theta _s)-\pi _s\theta _s)\, ds\right] , \end{aligned}$$

with $\pi \in \mathcal {A}(\mathbb {F})$. Using that the maximum is attained in the strategy given by (11), the solution of the optimal control problem is

28 $$\begin{aligned} \mathbb {V}^{\mathbb {F}}_T = \int _0^T \varvec{E}\left[ \rho _s - \frac{\mu _s-\rho _s}{\theta _s} + \lambda _s\ln \left( \frac{\lambda _s}{\lambda _s - (\mu _s-\rho _s)/{\theta _s}}\right) \right] ds, \end{aligned}$$

and by using (27) all the terms are well-defined. If $\pi \in \mathcal {A}(\mathbb {G})$, the Itô integral with respect to ${\tilde{N}}$ is not necessary well-defined, but by Lemma 3.4 we can still use it by taking advantage of the $\mathbb {G}$-semimartingale decomposition. We define the process

$$\begin{aligned} {\widehat{N}}_t := {\tilde{N}}_t - \int _0^t \lambda _s\gamma _s^G ds\ ,\quad 0\le t\le T, \end{aligned}$$

which is a $\mathbb {G}$-local martingale by Lemma 3.4. Then the dynamics of the wealth process satisfy the following SDE,

29 $$\begin{aligned} \frac{dX_t^\pi }{X_{t-}^{\pi }} = \left( (1-\pi _t)\rho _t+ \pi _t\mu _t+ \pi _t\theta _t\lambda _t\gamma _t^G \right) dt + \pi _t\theta _t d{\widehat{N}}_t ,\quad X_0 = x_0 , \end{aligned}$$

and we have the following explicit solution

$$\begin{aligned} \ln \frac{X_t^\pi }{x_0}&= \int _0^t\left( \rho _s + \pi _s(\mu _s-\rho _s) + \lambda _s(1+\gamma _s^G)\ln (1+\pi _s\theta _s)-\lambda _s\pi _s\theta _s\right) ds\\&\quad + \int _0^t \ln (1+\pi _s\theta _s) d{\widehat{N}}_s. \end{aligned}$$

As it is argued in Amendinger et al. (1998), by using the integrability condition of $\ln (1+\pi _t\theta _t)$, the stochastic integral satisfies

$$\begin{aligned} \varvec{E}\left[ \int _0^T \ln (1+\pi _s\theta _s)d {\widehat{N}}_s\right] = 0. \end{aligned}$$

In addition, for any $\mathbb {F}^N$-predictable $\beta =(\beta _t, \, 0 \le {t} \le {T})$,

30 $$\begin{aligned} 0 = \varvec{E}\left[ \int _0^t \beta _s d{\tilde{N}}_s - \int _0^t \beta _s d{{\widehat{N}}}_s\right] = \varvec{E}\left[ \int _0^t \beta _s\lambda _s\gamma _s^G ds \right] \ \end{aligned}$$

provided that $\varvec{E}\left[ \int _0^t |\beta _s| \lambda _s ds \right] <+\infty .$ Then,

31 $$\begin{aligned} \varvec{E}\left[ \ln \frac{X_T^\pi }{x_0}\right] = \int _0^T \varvec{E}\left[ \rho _s + \pi _s(\mu _s-\rho _s) + \lambda _s(1+\gamma _s^G)\ln (1+\pi _s\theta _s)-\lambda _s\pi _s\theta _s\right] ds. \end{aligned}$$

In the next proposition we compute the optimal strategy for a $\mathbb {G}$-agent.

#### Proposition 3.9

Let $G\in L^2(\varvec{P})$ be an $\mathcal {F}^N_T$-measurable random variable satisfying Assumption 3.1 with information drift $\gamma ^G$ verifying (20) and assume $\sigma _t = 0$, $\theta _t\ne 0$ $\textrm{d}t\times \textrm{d}\varvec{P}$ a.s. Then, the optimal portfolio $\pi ^G$ solving the problem (8) with information flow $\mathbb {G}$ is given by

32 $$\begin{aligned} \pi _t^G = \frac{\mu _t-\rho _t}{\lambda _t\theta _t^2-\theta _t(\mu _t-\rho _t)} + \frac{\lambda _t\gamma ^G_t}{\lambda _t\theta _t-(\mu _t-\rho _t)}. \end{aligned}$$

#### Proof

We refer to the “Appendix A” for the details of the proof. $\square$

#### Remark 3.10

Note that the strategy (32) satisfies the admissibility condition (7) thanks to the assumptions (20) and (27). In particular,

33 $$\begin{aligned} 1+\pi _t^G\theta _t&= 1 + \frac{\mu _t-\rho _t}{\lambda _t\theta _t-(\mu _t-\rho _t)} + \frac{\lambda _t\theta _t\gamma ^G_t}{\lambda _t\theta _t-(\mu _t-\rho _t)}\nonumber \\&= \frac{\lambda _t\theta _t(1+\gamma ^G_t)}{\lambda _t\theta _t-(\mu _t-\rho _t)} = \frac{\lambda _t(1+\gamma ^G_t)}{\lambda _t-(\mu _t-\rho _t)/\theta _t} > 0 \quad \textrm{d}t\times \textrm{d}\varvec{P}-a.s. \end{aligned}$$

The next theorem is one of the main results as it gives the difference of the expected gains under logarithmic utility in a pure jump market of the additional information $G\in \mathcal {F}_T^N$.

#### Theorem 3.11

Let $\mathbb {G}\supset \mathbb {F}$ be the initial enlargement with $G\in L^2(\varvec{P})$ an $\mathcal {F}_T^N$-measurable random variable satisfying Assumption 3.1 with information drift $\gamma ^G$ verifying (20), then

34 $$\begin{aligned} {\Delta \mathbb {V}_T^\mathbb {G}} = \int _0^T \varvec{E}\left[ \lambda _s(1+\gamma _s^G) \ln \left( \lambda _s(1+\gamma _s^G) \right) -\lambda _s\ln \lambda _s\right] ds \ge 0 . \end{aligned}$$

#### Proof

We refer to the “Appendix A” for the details of the proof. $\square$

## Mixed Brownian–Poisson market

We extend the results of Sect. 3 to the case of Brownian–Poisson market, i.e., the dynamics of the risky asset are driven by a Brownian motion and a compensated Poisson process and the market coefficients satisfy $\sigma _t,\theta _t\ne 0$ ${\textrm{d}t\times \textrm{d}\varvec{P}}$-a.s. We also maintain the assumption that $\lambda$ is deterministic in this section. Using the Clark–Ocone formula stated in (13), we can apply the same representation formula for every $\mathcal {F}_T$-measurable random variable $G\in L^2(\varvec{P})$,

$$\begin{aligned} G = \varvec{E}[G] + \int _0^T \phi _s d W_s + \int _0^T \varphi _s d{\tilde{N}}_s, \end{aligned}$$

where the processes $\phi$ and $\varphi$ are related to the Malliavin derivative. Let $B\in \mathcal {B}(\mathbb {R})$ be a subset and we consider the following PRP

35 $$\begin{aligned} \mathbbm {1}_{\{G\in B\}} = \varvec{P}^G(B) + \int _0^T \phi _s(B)dW_s + \int _0^T \varphi _s(B)d{\tilde{N}}_s , \end{aligned}$$

given in Theorem 13.28 of Di Nunno et al. (2009). As before, we shall make the following assumptions.

### Assumption 4.1

The vector measures $\phi ,\varphi$ are of bounded variation.

If we reason as in Remark 3.2, we can prove that the previous Assumption 4.1 is satisfied for discrete random variables.

We fix *g* in the support of *G*, by reasoning as in the beginning of Sect. 3, see Lemma 3.3, we conclude that there exist two $\mathbb {F}$-adapted processes $\zeta ^g,\,\psi ^g$ with $g\in {{\,\textrm{Supp}\,}}(G)$ such that the following PRP

$$\begin{aligned} \mathbbm {1}_{\{G\in B\}} = \varvec{P}^G(B)+ \int _0^T\int _B \zeta ^g_s \varvec{P}^G(dg) dW_s + \int _0^T\int _B \psi ^{g}_s \varvec{P}^G(dg) d{\tilde{N}}_s. \end{aligned}$$

Provided the measurability of $\zeta$ and $\psi$ in all variables, we can write the following representation

36 $$\begin{aligned} \mathbbm {1}_{\{G\in dg\}} = \varvec{P}^G(dg)+ \int _0^T \zeta ^g_sdW_s\,\varvec{P}^G(dg) + \int _0^T \psi ^{g}_s d{\tilde{N}}_s\,\varvec{P}^G(dg) , \end{aligned}$$

with $\phi ^{g}_t=\zeta _t^g\varvec{P}(G=g)$ and $\varphi ^{g}_t=\psi _t^g\varvec{P}(G=g)$ in (36). when *G* is purely atomic. Note that, by using our standing assumptions, we have achieved a representation usually assumed in the literature, see Proposition 4 of Wright et al. (2018) for the Poisson case.

### Lemma 4.2

Let $G\in L^2(\varvec{P})$ be an $\mathcal {F}_T$-measurable random variable satisfying the Assumption 4.1, then the processes $\alpha ^G$ and $\gamma ^G$ defined as

37 $$\begin{aligned} \alpha _t^g := \frac{\zeta ^g_t\varvec{P}^G(dg)}{\varvec{P}^G(dg\vert \mathcal {F}_t)}\ ,\quad \gamma _t^g := \frac{\psi ^g_t\varvec{P}^G(dg)}{\varvec{P}^G(dg\vert \mathcal {F}_t)} ,\quad 0\le t < T , \end{aligned}$$

satisfy that $W_{\cdot }-\int _0^{\cdot } \alpha ^G_s ds$ and $\tilde{N}_{\cdot } - \int _0^{\cdot }\lambda _s\gamma _s^G ds$ are $\mathbb {G}$-local martingales, provided they are well-defined.

### Proof

Taking into account that $[W,{\tilde{N}}]_t^\mathbb {F}= 0$, the proof of the following lemma follows from the same lines as the proof of Lemma 3.4. Let $A\in \mathcal {F}_s$ and $B\in \sigma (G)$, then

$$\begin{aligned}&\varvec{E}[\mathbbm {1}_A\mathbbm {1}_{\{G\in B\}}({\tilde{N}}_t-{\tilde{N}}_s)] \\&\quad = \varvec{E}\left[ \mathbbm {1}_A\left( \varvec{P}^G(B)+ \int _0^T\int _B \zeta ^g_s \varvec{P}^G(dg) dW_s + \int _0^T \int _B \psi ^{g}_s \varvec{P}^G(dg) d{\tilde{N}}_s\right) ({\tilde{N}}_t-{\tilde{N}}_s)\right] \\&\quad = \varvec{E}\left[ \mathbbm {1}_A \int _s^t\int _B \psi ^g_u \varvec{P}^G(dg) dN_u \right] =\varvec{E}\left[ \mathbbm {1}_A \int _s^t\int _B \lambda _u\gamma _u^g\varvec{P}^G(dg\vert \mathcal {F}_u) du \right] \\&\quad = \varvec{E}\left[ \mathbbm {1}_A\mathbbm {1}_{\{G\in B\}} \int _s^t\lambda _u\gamma _u^Gdu \right] , \end{aligned}$$

and the result follows true by mimicking the computation for the Brownian motion *W*. $\square$

As in the previous section, we keep assuming

$$\gamma _s^G>-1\, \textrm{d}t\times \textrm{d}\varvec{P}\text {-}a.s.$$

in order to maintain that the stochastic $\mathbb {G}$-intensity $\lambda (1+\gamma ^G)$ is positive.

### Corollary 4.3

If $${{\,\textrm{Supp}\,}}(G)=\{0,1,\ldots ,n\}$$, then

$$\begin{aligned} \alpha _t^g = \phi ^g_t \frac{\mathbbm {1}_{\{G=g\}} -\varvec{E}[\mathbbm {1}_{\{G=g\}}\vert \mathcal {F}_t]}{\varvec{V}[\mathbbm {1}_{\{G=g\}}\vert \mathcal {F}_t]}\ ,\quad \gamma _t^g = \varphi ^g_t \frac{\mathbbm {1}_{\{G=g\}} -\varvec{E}[\mathbbm {1}_{\{G=g\}}\vert \mathcal {F}_t]}{\varvec{V}[\mathbbm {1}_{\{G=g\}}\vert \mathcal {F}_t]} , \end{aligned}$$

where we consider $\phi ^g_t = \zeta ^g_t\varvec{P}(G=g)$ and $\varphi ^g_t = \psi ^g_t\varvec{P}(G=g)$ with $g\in {{\,\textrm{Supp}\,}}(G)$. In addition, if ${{\,\textrm{Supp}\,}}(G)=\{0,1\}$, then

38 $$\begin{aligned} \alpha _t^G = (-1)^{G+1}\phi _t \frac{G-\varvec{E}[G\vert \mathcal {F}_t]}{\varvec{V}[G\vert \mathcal {F}_t]} ,\quad \gamma _t^G = (-1)^{G+1}\varphi _t\frac{G-\varvec{E}[G\vert \mathcal {F}_t]}{\varvec{V}[G\vert \mathcal {F}_t]}. \end{aligned}$$

When we consider a $\mathbb {G}$-agent playing with $\pi \in \mathcal {A}(\mathbb {G})$, the Itô integral fails for both the Brownian motion and the Poisson process. Using Lemma 4.2 we can define the following $\mathbb {G}$-local martingales

39 $$\begin{aligned} {\widehat{W}}_t:= W_t-\int _0^t \alpha ^G_s ds ,\quad {\widehat{N}}_t:= {\tilde{N}}_t - \int _0^t \lambda _s\gamma _s^Gds ,\quad 0\le t \le T\ . \end{aligned}$$

The dynamics of the wealth process satisfy the following SDE,

40 $$\begin{aligned} \frac{dX_t^\pi }{X_{t-}^{\pi }} = \left( (1-\pi _t)\rho _t+ \pi _t\mu _t + \pi _t\sigma _t\alpha _t^G+ \pi _t\theta _t\lambda _t\gamma _t^G \right) dt + \pi _t\sigma _t d{\widehat{W}}_t + \pi _t\theta _t d{\widehat{N}}_t. \end{aligned}$$

To short the notation, we define the following terms

$$\begin{aligned} \begin{array}{ll} d^G_s := \mu _s-\rho _s+\alpha _s^G\sigma _s-\lambda _s\theta _s , &{} c^G_s := \lambda _s(1+\gamma ^G_s) ,\\ d_s := \mu _s-\rho _s-\lambda _s\theta _s , &{}c_s :=\lambda _s . \end{array} \end{aligned}$$

Finally, using the integrability conditions, we can compute the expectation of the stochastic integrals and we get,

$$\begin{aligned} \varvec{E}\left[ \ln \frac{X^{\pi }_T}{x_0}\right]&= \int _0^{T}\varvec{E}\Big [ \rho _s + \pi _s d^G_s - \frac{1}{2}\pi ^2_s\sigma ^2_s + c^G_s \ln (1+\pi _s\theta _s)-\lambda _s\pi _s\theta _s \Big ]ds. \end{aligned}$$

### Proposition 4.4

The optimal strategy of the problem (8) with $G\in L^2(\varvec{P})$ being an $\mathcal {F}_T$-measurable random variable satisfying Assumption 4.1 such that the information drift $\gamma ^G$ verifies (20), information flow $\mathbb {G}$ and both Brownian and Poisson noises is given by,

41 $$\begin{aligned} {\pi _s = \frac{1}{2}\left( \frac{d^G_s }{\sigma _s^2}-\frac{1}{\theta _s} \right) +{{\,\textrm{sgn}\,}}(\theta _s)\frac{1}{2} \sqrt{\left( \frac{d^G_s }{\sigma _s^2}+\frac{1}{\theta _s}\right) ^2+ 4\frac{c^G_s}{\sigma _s^2}} ,\quad 0\le s \le T. } \end{aligned}$$

### Proof

We refer to the “Appendix A” for the details of the proof. $\square$

### Remark 4.5

If we denote by $\pi _s^W := (\mu _s-\rho _s+\alpha ^G_s\sigma _s)/\sigma ^2_s$ the optimal strategy for the only Brownian market with additional information then we can write the general strategy for the Poisson-Brownian mixed market as

$$\begin{aligned} \pi _s = \frac{1}{2}\left( \pi _s^W-\lambda _s\frac{\theta _s}{\sigma ^2_s}-\frac{1}{\theta _s} \right) + {{\,\textrm{sgn}\,}}(\theta _s)\frac{1}{2} \sqrt{\left( \pi _s^W-\lambda _s\frac{\theta _s}{\sigma ^2_s}+\frac{1}{\theta _s}\right) ^2+ 4\lambda _s\frac{1+ \gamma ^G_s}{\sigma _s^2}}. \end{aligned}$$

The expected logarithmic gains under $\mathbb {G}$ are computed in the following theorem.

### Theorem 4.6

Let $\mathbb {G}\supset \mathbb {F}$ be the initial enlargement with $G\in L^2(\varvec{P})$ being an $\mathcal {F}_T$-measurable random variable satisfying Assumption 4.1 such that the information drift $\gamma ^G$ verifies (20), then

42 $$\begin{aligned} \mathbb {V}_T^\mathbb {G}&= \int _0^T\varvec{E}\Bigg [ \frac{1}{2} \left( \frac{d^G_s}{\sigma _s}\right) ^2+ \frac{1}{2}\frac{d^G_s}{\sigma _s}{{\,\textrm{sgn}\,}}(\theta _s)\sqrt{\left( \frac{d^G_s}{\sigma _s}+\frac{\sigma _s}{\theta _s}\right) ^2+4c^G_s} -\frac{1}{2}\frac{d_s}{\theta _s} +c^G_s\ln c^G_s \nonumber \\&\quad +\lambda _s \ln \left( 2\frac{\theta _s^2}{\sigma _s^2}\right) - c^G_s\ln \left( \sqrt{\left( \frac{d^G_s\theta _s}{\sigma _s^2} + 1 \right) ^2 + 4\frac{\theta _s^2}{\sigma _s^2}c^G_s} - \left( \frac{d^G_s\theta _s}{\sigma _s^2} + 1 \right) \right) \Bigg ] ds . \end{aligned}$$

### Proof

We refer to the “Appendix A” for the details of the proof. $\square$

### Corollary 4.7

Under the set-up of Theorem 4.6,

43 $$\begin{aligned} \Delta \mathbb {V}_T^\mathbb {G}&= \int _0^T\varvec{E}\left[ \frac{1}{2} \left( \alpha ^G_s\right) ^2 +c^G_s\ln c^G_s-\lambda _s\ln \lambda _s + f_s^{G}-f_s+ h_s-h_s^{G}\right] ds \end{aligned}$$

where we define the following terms,

$$\begin{aligned} f_s^{(G)}&:= \frac{1}{2}\left( \frac{d^{(G)}_s}{\sigma _s}\right) {{\,\textrm{sgn}\,}}(\theta _s)\sqrt{\left( \frac{d^{(G)}_s}{\sigma _s}+\frac{\sigma _s}{\theta _s}\right) ^2+4c^{(G)}_s}\\ h_s^{(G)}&:= c^{(G)}_s\ln \left( \sqrt{\left( \frac{d^{(G)}_s\theta _s}{\sigma _s^2} + 1 \right) ^2 + 4\frac{\theta _s^2}{\sigma _s^2}c^{(G)}_s} - \left( \frac{d^{(G)}_s\theta _s}{\sigma _s^2} + 1 \right) \right) . \end{aligned}$$

### Remark 4.8

Corollary 4.7 can be seen as a generalization of the additional expected logarithmic utility in Amendinger et al. (1998) for the case of market with jumps. If we assume that the information is only related to the Brownian motion, i.e., $\gamma ^G = 0$, the additional gains can be easily computed. For the sake of simplicity, we also choose the market coefficients as $\rho =\mu$, $\lambda =\sigma = 1$ and $\theta _s\in \{-a,a\}$, for any $a\in (0,1)$, and we obtain

44 $$\begin{aligned} \mathbb {V}_T^{\mathbb {G}}-\mathbb {V}_T^{\mathbb {F}}&= \int _0^T\varvec{E}\left[ \frac{1}{2} \left( \alpha ^G_s\right) ^2 + \frac{a}{2}\sqrt{\left( 1-\frac{1}{a}\right) ^2+4} \right. \nonumber \\&\quad \left. + \frac{1}{2}\left( \frac{\alpha _s^G\theta _s-a^2}{a}\right) \sqrt{\left( \frac{\alpha _s^G\theta _s-a^2}{a} + \frac{1}{\theta _s}\right) ^2+4} + \ln 2a^2 \right. \nonumber \\&\quad \left. - \ln \left( \sqrt{\left( \alpha _s^G\theta _s +1-a^2\right) ^2 + 4a^2} - \alpha _s^G\theta _s -1+a^2\right) \right] ds. \end{aligned}$$

Accordingly, the additional expected logarithmic utility in the mixed-market when the information is only related to the Poisson process, i.e. $\alpha ^G=0$, is

45 $$\begin{aligned} \mathbb {V}_T^{\mathbb {G}}-\mathbb {V}_T^{\mathbb {F}}&= \int _0^T\varvec{E}\left[ (1+\gamma _s^G)\ln \left( 1+\gamma _s^G\right) + \frac{a}{2}\sqrt{\left( 1-\frac{1}{a}\right) ^2+4} \right. \nonumber \\&\quad \left. - \frac{a}{2}\sqrt{\left( -a + \frac{1}{\theta _s}\right) ^2+4(1+\gamma _s^G)} + \ln 2a^2 \right. \nonumber \\&\quad \left. - (1+\gamma _s^G)\ln \left( \sqrt{\left( 1+a^2\right) ^2 + 4a^2\gamma _s^G}-1+a^2\right) \right] ds , \end{aligned}$$

under the same simplification of the market coefficients.

### Example 4.9

Let $A = (-\infty ,a]$ and $B=(-\infty ,b]$ be two half-bounded intervals. We define the Bernoulli random variable as the following product indicator,

$$\begin{aligned} G = \mathbbm {1}_{\{W_T\le a\}\times \{N_T\le b\}} = \mathbbm {1}_{\{W_T\le a\}}\mathbbm {1}_{\{N_T\le b\}}. \end{aligned}$$

According to León et al. (2002), thanks to the independence of the Brownian motion and the Poisson process, the Malliavin derivatives in each direction can be easily computed as follows,

$$\begin{aligned} D_t G = \mathbbm {1}_{\{N_T\le b\}} D_t \mathbbm {1}_{\{W_T\le a\}} ,\quad D_{t,1} G = \mathbbm {1}_{\{W_T\le a\}} D_{t,1} \mathbbm {1}_{\{N_T\le b\}} \end{aligned}$$

so the conditional expectations are calculated as follows,

$$\begin{aligned} \varvec{E}\left[ D_t G \vert \mathcal {F}_t\right]&= \varvec{E}\left[ \mathbbm {1}_{\{N_T\le b\}} D_t \mathbbm {1}_{\{W_T\le a\}}\vert \mathcal {F}_t\right] = {-}\varvec{E}\left[ \mathbbm {1}_{\{N_T\le b\}} \delta _a(W_T)\vert \mathcal {F}_t\right] , \end{aligned}$$

where we refer to Section 5 of Bermin (2002) for the generalized Malliavin derivative of the indicator function and the definition of the Dirac delta $\delta _a(\cdot )$. In order to compute the conditional expectation, we consider the following conditional distribution function,

$$\begin{aligned} F(x,y):=\varvec{P}(W_T\le x,N_T\le y\vert \mathcal {F}_t) = \int _{-\infty }^x \sum _{k = 0}^y f_{W_T\vert W_t}(u) p_{N_T\vert N_t}(k) du, \end{aligned}$$

where $f_{W_T\vert W_t}(u)$ denotes the density function of $(W_T\vert W_t)$ and $p_{N_T\vert N_t}(k)$ the probability function of $(N_T\vert N_t)$. Both of them are well-known, then,

$$\begin{aligned} \varvec{E}\left[ D_t G \vert \mathcal {F}_t\right]&= {-} \int _{-\infty }^\infty \sum _{k = 0}^\infty f_{W_T\vert W_t}(u) p_{N_T\vert N_t}(k) \mathbbm {1}_{\{k\in B\}} \delta _a(u) du\\&= {-}f_{W_T\vert W_t}(a) \sum _{k = 0}^b p_{N_T\vert N_t}(k)\\&={-} \frac{\exp \left( -\frac{(a-W_t)^2}{2(T-t)}\right) }{\sqrt{2\pi (T-t)}} \sum _{k = 0}^{b-N_t} e^{-\Lambda (t,T)}\frac{(\Lambda (t,T))^k}{k!}\mathbbm {1}_{\{N_t\le b\}}. \end{aligned}$$

With respect to the Poisson component, we compute the conditional expectation of $D_{t,1}G$ as follows,

$$\begin{aligned} \varvec{E}\left[ D_{t,1} G\vert \mathcal {F}_t\right]&= \varvec{E}\left[ \mathbbm {1}_{\{W_T\le a\}} D_{t,1} \mathbbm {1}_{\{N_T\le b\}}\vert \mathcal {F}_t\right] = - \varvec{E}\left[ \mathbbm {1}_{\{W_T\le a\}}\mathbbm {1}_{\{N_T = b\}}\vert \mathcal {F}_t\right] \\&= - \varvec{E}\left[ \mathbbm {1}_{\{W_T\le a\}\times \{N_T = b\}}\vert \mathcal {F}_t\right] = - \varvec{P}\left( W_T\le a,\, N_T = b\vert \mathcal {F}_t\right) \\&= -\left( \int _{-\infty }^{a}\frac{\exp \left( -\frac{(x-W_t)^2}{2(T-t)}\right) }{\sqrt{2\pi (T-t)}} dx\right) \left( e^{-\Lambda (t,T)}\frac{(\Lambda (t,T))^{b-N_t}}{(b-N_t)!} \mathbbm {1}_{\{N_t\le b\}}\right) \end{aligned}$$

where we have used our Example 3.8, with $b_1 = 0,\,b_2=b$, in order to compute the Malliavin derivative. Then, the processes $\alpha ^G$ and $\gamma ^G$ appearing in (38) are determined. Finally, we deduce the PRP via Clark–Ocone formula,

$$\begin{aligned}&\mathbbm {1}_{\{W_T\le a\}\times \{N_T\le b\}} = \varvec{P}(W_T\le a)\varvec{P}(N_T\le b) \\&{-} \int _0^T \left( \frac{\exp \left( -\frac{(a-W_t)^2}{2(T-t)}\right) }{\sqrt{2\pi (T-t)}}\right) \left( \sum _{k = 0}^{b-N_t} e^{-\Lambda (t,T)}\frac{(\Lambda (t,T))^k}{k!} \right) dW_t\\&-\int _0^T\left( \int _{-\infty }^{a-W_t}\frac{\exp \left( -\frac{(x-a+W_t)^2}{2(T-t)}\right) }{\sqrt{2\pi (T-t)}} dx\right) \left( e^{-\Lambda (t,T)}\frac{(\Lambda (t,T))^{b-N_t}}{(b-N_t)!} \mathbbm {1}_{\{N_t\le b\}}\right) d{\tilde{N}}_t. \end{aligned}$$

### Example 4.10

Let’s define

46 $$\begin{aligned} M_{s,t}:=\sup _{s\le u \le t} W_u ,\quad J_{s,t}:=\sup _{s\le u \le t} {\tilde{N}}_u , \end{aligned}$$

and $M_t:= M_{0,t}$ and $J_t:= J_{0,t}$. To short the notation, we define the intervals $A:=(a_1,a_2]$ and $B=(b_1,b_2]$. We consider the following example

47 $$\begin{aligned} G = \mathbbm {1}_{\{M_T\in A\}\times \{J_T\in B\}} = \mathbbm {1}_{\{M_T\in A\}}\mathbbm {1}_{\{J_T\in B\}} \end{aligned}$$

and we proceed as before.

$$\begin{aligned} D_t G&= \mathbbm {1}_{\{J_T\in B\}} D_t \mathbbm {1}_{\{M_T\in A\}} = \mathbbm {1}_{\{J_T\in B\}} D_t \left( \mathbbm {1}_{\{M_T\le a_2\}} - \mathbbm {1}_{\{M_T \le a_1\}}\right) \\&= \mathbbm {1}_{\{J_T\in B\}} \mathbbm {1}_{\{M_t\le M_{t,T}\}}\left( -\delta _{a_2}(M_T) + \delta _{a_1}(M_T)\right) , \end{aligned}$$

we refer to Bermin (2002) for a detailed explanation of the Malliavin derivative of the running maximum $M_T$. Thanks to the independence, we restrict our analysis to the following term

48 $$\begin{aligned} \varvec{E}\left[ D_t \mathbbm {1}_{\{M_T\in A\}} \vert \mathcal {F}_t\right]&= \varvec{E}\left[ \mathbbm {1}_{\{M_t\le M_{t,T}\}}\left( -\delta _{a_2}(M_{t,T}) + \delta _{a_1}(M_{t,T})\right) \vert \mathcal {F}_t\right] \nonumber \\&= \int _0^{+\infty } \mathbbm {1}_{\{M_t\le m\}}\left( -\delta _{a_2}(m) + \delta _{a_1}(m)\right) f^M_{t}(m) dm\nonumber \\&= \mathbbm {1}_{\{M_t\le a_1\}} f^M_{t}(a_1) - \mathbbm {1}_{\{M_t\le a_2\}} f^M_{t}(a_2) , \end{aligned}$$

being $f^M_t$ the density of the random variable $M_{t,T}$ given $\mathcal {F}_t$, which is equivalent to consider the variable $M_{T-t}$ in the domain $(W_t,+\infty )$, i.e.,

$$\begin{aligned} f^M_t(m) =\frac{2 e^{-\frac{(m-W_t)^2}{2(T-t)}}}{ \sqrt{2\pi (T-t)}} ,\quad m\ge W_t. \end{aligned}$$

On the other hand we compute the conditional expectation of the remained Poisson term,

$$\begin{aligned} \varvec{E}\left[ \mathbbm {1}_{\{J_T\in B\}}\vert \mathcal {F}_t\right]&= \varvec{P}\left( J_T\in B\vert \mathcal {F}_t\right) = \varvec{P}\left( \max \{J_t,J_{t,T}\}\in B\vert \mathcal {F}_t\right) \\&= \varvec{P}\left( J_t + (J_{t,T}-J_t)^+\in B\vert \mathcal {F}_t\right) \\&= \varvec{P}\left( (J_{T-t}-b_t)^+\in (b_1-j,b_2-j] \right) {\vert _{j=J_t}} , \end{aligned}$$

where $b_t:= j-{\tilde{N}}_t$ and using that $J_{t,T}-{\tilde{N}}_t$ is independent of $\mathcal {F}_t$. We aim to compute

49 $$\begin{aligned} \varvec{E}\left[ \mathbbm {1}_{\{J_T\in B\}}\vert \mathcal {F}_t\right]&= \varvec{P}\left( (J_{T-t}-b_t)^+> b_1-j\right) {\vert _{j=J_t}}\nonumber \\&\quad - \varvec{P}\left( (J_{T-t}-b_t)^+ > b_2-j\right) {\vert _{j=J_t}}. \end{aligned}$$

Each one of the probabilities can be computed as

$$\begin{aligned} \varvec{P}\left( (J_{T-t}-b_t)^+> b_1-j\right) {\vert _{j=J_t}}&= \mathbbm {1}_{\{b_1 - J_t< 0\}} + \mathbbm {1}_{\{b_1 - J_t \ge 0\}}\overline{F}^N_{T-t}(b_1-{\tilde{N}}_t) \\&= 1+ \mathbbm {1}_{\{b_1 - J_t \ge 0\}}\left( \overline{F}^N_{T-t}(b_1-{\tilde{N}}_t)-1\right) \\ \varvec{P}\left( (J_{T-t}-b_t)^+ > b_2-j\right) {\vert _{j=J_t}}&= \mathbbm {1}_{\{b_2 - J_t < 0\}} + \mathbbm {1}_{\{b_2 - J_t \ge 0\}}\overline{F}^N_{T-t}(b_2-{\tilde{N}}_t)\\&= 1+ \mathbbm {1}_{\{b_2 - J_t \ge 0\}}\left( \overline{F}^N_{T-t}(b_2-{\tilde{N}}_t)-1\right) \end{aligned}$$

where the survival function is defined as $\overline{F}^N_{T-t}(x) = \varvec{P}(J_{T-t}>x)$ for every $x\ge 0$. See Kuznetsov (2010) for an explicit computation of the distribution of the running supremum. In terms of the distribution function $F^N$, (49) can be simplified as follows,

50 $$\begin{aligned} \varvec{E}\left[ \mathbbm {1}_{\{J_T\in B\}}\vert \mathcal {F}_t\right]&= \mathbbm {1}_{\{b_1 - J_t \ge 0\}}\left( \overline{F}^N_{T-t}(b_1-{\tilde{N}}_t)-1\right) \nonumber \\&\quad - \mathbbm {1}_{\{b_2 - J_t \ge 0\}}\left( \overline{F}^N_{T-t}(b_2-{\tilde{N}}_t)-1\right) \nonumber \\&= \mathbbm {1}_{\{b_2 - J_t \ge 0\}}F^N_{T-t}(b_2-\tilde{N}_t)-\mathbbm {1}_{\{b_1 - J_t \ge 0\}}F^N_{T-t}(b_1-{\tilde{N}}_t). \end{aligned}$$

Then, taking into account (48) and (50) the process $\alpha ^G$ is fully determined by (38). We proceed in the same way in order to compute $\varvec{E}[D_{t,1}G\vert \mathcal {F}_t]$. Using the operator $\Psi$ given in (23), we can compute the following Malliavin derivative

$$\begin{aligned} D_{t,1} \mathbbm {1}_{\{J_T\in B\}} =\mathbbm {1}_{\{\max {\{J_t,1+J_{t,T}\}}\in B\}} - \mathbbm {1}_{\{J_T\in B\}} \end{aligned}$$

where the second term has been calculated in (50). For the first one we have

$$\begin{aligned} \varvec{E}[\mathbbm {1}_{\{\max {\{J_t,1+J_{t,T}\}}\in B\}}\vert \mathcal {F}_t]&= \varvec{P}(\max {\{J_t,1+J_{t,T}\}}\in B\vert \mathcal {F}_t) \\&= \mathbbm {1}_{\{b_2 - J_t \ge 0\}}F^N_{T-t}(b_2-{\tilde{N}}_t-1)\\&\quad -\mathbbm {1}_{\{b_1 - J_t \ge 0\}}F^N_{T-t}(b_1-{\tilde{N}}_t-1) \end{aligned}$$

where we have omitted some steps as they were similar to ones shown before. Finally

51 $$\begin{aligned} \varvec{E}\left[ \mathbbm {1}_{\{M_T\in A\}}\vert \mathcal {F}_t\right] = \mathbbm {1}_{\{a_2 - M_t \ge 0\}}F^W_{T-t}(a_2- W_t)-\mathbbm {1}_{\{a_1 - M_t \ge 0\}}F^W_{T-t}(a_1- W_t) , \end{aligned}$$

where in this case $\overline{F}_t^W(y) = 2(1-\Phi (y/\sqrt{t}))$ and again the process $\gamma ^G$ is determined by (38).

## Stochastic intensity in Brownian–Poisson market

In this section, we assume that ${\tilde{N}}$ is an inhomogeneous Poisson process with stochastic intensity. We introduce $\mathcal {F}^\lambda _t:=\sigma (\lambda _s,s\le t)$ and we assume that $\mathcal {F}_T^\lambda \subset \mathcal {F}_T$. We will look at examples where $\mathcal {F}_T^\lambda \subset \mathcal {F}^W_T$, allowing some dependence of $\lambda$ with respect to *W*, however more general cases may be considered, we prefer not to include them for easy of presentation. In particular, following Section 8.4.2 of Jeanblanc et al. (2009), we assume that

$$\begin{aligned} \varvec{E}\left[ \int _0^T \phi _sdN_s\right] = \varvec{E}\left[ \int _0^T \phi _s\lambda _sds\right] , \end{aligned}$$

being $\phi$ any $\mathbb {F}\vee \mathbb {F}^\lambda$-adapted process and $\tilde{N}_\cdot = N_\cdot - \int _0^\cdot \lambda _sds$ is an $\mathbb {F}\vee \mathbb {F}^\lambda$-martingale. The standing assumption of the section is that *G* satisfies the following representation for any $B\in \sigma (G)$.

### Assumption 5.1

52 $$\begin{aligned} \mathbbm {1}_{\{G\in B\}} = \varvec{P}^G(B\vert \mathcal {F}_T^\lambda )+ \int _0^T \int _B\zeta ^g_s \varvec{P}^G(dg) dW_s + \int _0^T\int _B \psi ^{g}_s \varvec{P}^G(dg) d{\tilde{N}}_s , \end{aligned}$$

and there exist some $\mathbb {F}\vee \mathcal {F}_T^\lambda$-adapted processes ${\bar{\alpha }}^\lambda$ and ${\bar{\gamma }}^\lambda$ such that $W_\cdot -\int _0^\cdot {\bar{\alpha }}^\lambda _sds$ and ${\tilde{N}}_\cdot - \int _0^\cdot {\bar{\gamma }}^\lambda _s ds$ are $\mathbb {F}\vee \mathcal {F}_T^\lambda$-martingales, provided $\zeta$ and $\psi$ are as usual measurable in all variables.

From the Assumption 5.1 we conclude that with ${\widehat{\alpha }}^\lambda _t:=\varvec{E}[{\bar{\alpha }}^\lambda _t\vert \mathcal {F}_t \vee \mathcal {F}_t^\lambda ]$, $0\le t \le T$, the process $W_\cdot -\int _0^\cdot {\widehat{\alpha }}^\lambda _sds$ is an $\mathbb {F}\vee \mathbb {F}^\lambda$-martingale.

Next proposition gives the equivalent of Lemma 4.2 for the case of $\lambda$ not adapted to the Poisson filtration. Note that additional terms appear in the information drifts induced by the unpredictability of $\lambda$.

### Proposition 5.2

Let $G\in L^2(\varvec{P})$ be an $\mathcal {F}_T$-measurable random variable satisfying Assumption 5.1 and suppose that $\mathbb {F}\vee \mathbb {F}^\lambda \subset \mathbb {G}$ holds. Then, for any $(t,g)\in [0,T)\times {{\,\textrm{Supp}\,}}(G)$, we define

$$\begin{aligned}{} & {} \alpha _t^g := \frac{\zeta ^g_t\varvec{P}^G(dg)}{\varvec{P}^G(dg\vert \mathcal {F}_t)} ,\quad {{\widetilde{\alpha }}}^\lambda _t :=\frac{{\bar{\alpha }}_t^\lambda \varvec{P}^G(dg\vert \mathcal {F}^\lambda _T)}{\varvec{P}^G(dg\vert \mathcal {F}_t \vee \mathcal {F}^\lambda _T)} ,\\{} & {} \gamma _t^g := \frac{\psi ^{g}_t\varvec{P}^G(dg)}{\varvec{P}^G(dg\vert \mathcal {F}_t)} ,\quad {{\widetilde{\gamma }}}^\lambda _t :=\frac{{\bar{\gamma }}_t^\lambda \varvec{P}^G(dg\vert \mathcal {F}^\lambda _T)}{\varvec{P}^G(dg\vert \mathcal {F}_t \vee \mathcal {F}^\lambda _T)} ,\quad {\widehat{\gamma }}^g_t := \frac{\zeta _t^g{\widehat{\alpha }}_t^\lambda \varvec{P}^G(dg)}{\varvec{P}^G(dg\vert \mathcal {F}_t\vee \mathcal {F}^\lambda _t)} , \end{aligned}$$

such that the processes

$$\begin{aligned} W_{\cdot }&-\int _0^{\cdot } \left( \alpha ^G_s + {{\widetilde{\alpha }}}^\lambda _s \right) ds ,\\ N_\cdot&- \int _0^\cdot \left( \lambda _v +{{\widetilde{\gamma }}}_v^\lambda + \lambda _v\gamma ^G_v -\lambda _v \varvec{E}\left[ \int _v^T{\widehat{\gamma }}^G_udu\vert \mathcal {G}_v\right] \right) dv \end{aligned}$$

are $\mathbb {G}$-local martingales, provided the integrands, i.e. the information drifts, are well-defined.

### Proof

We refer to the “Appendix A” for the details of the proof. $\square$

### Example 5.3

We consider a stochastic intensity given by

$$\begin{aligned} \lambda _t = 1 +\mathbbm {1}_{\{W_T\ge 0\}} ,\quad 0\le t\le T. \end{aligned}$$

We introduce $G=\mathbbm {1}_{\{W_T\ge 0\}}$ and note that $\mathcal {F}_t^\lambda = \mathcal {F}_T^\lambda = \sigma (G)$, for any $0\le t\le T$ and we conclude that ${\widehat{\alpha }}^\lambda ={\bar{\alpha }}^\lambda ={{\widetilde{\alpha }}}^\lambda$ and ${{\widetilde{\gamma }}}^\lambda ={\bar{\gamma }}=0$. Let’s define the initial enlargement $\mathbb {G}=\mathbb {F}\vee \sigma (G)$. Then the representation (5.1) is reduced to

$$\begin{aligned} G = \varvec{E}[G\vert \mathcal {F}_T^\lambda ] \end{aligned}$$

so we conclude that $\zeta ^g = \psi ^g = 0$ and then $\gamma ^G = {\widehat{\gamma }}^G = \alpha ^G = 0$. We use the well-known PRP

$$\begin{aligned} \mathbbm {1}_{\{ W_T\ge 0 \}} = \varvec{P}( W_T\ge 0) + \int _0^T \frac{1}{\sqrt{2\pi (T-t)}}\exp \left( -\frac{W_t^2}{2(T-t)}\right) dW_t , \end{aligned}$$

from which we conclude, see Proposition 4.7 in D’Auria and Salmerón (2020) for details, that

$$\begin{aligned} {{\widetilde{\alpha }}}^\lambda _t = \frac{(-1)^{\lambda }}{\sqrt{2\pi (T-t)}}\exp \left( -\frac{W_t^2}{2(T-t)}\right) \ ,\quad \lambda \in \{1,2\} , \end{aligned}$$

and the information drifts appearing in Proposition 5.2 are determined. By concatenating the information drifts, the technique can be easily generalized to the case when

$$\begin{aligned} \lambda _t = 1 +\mathbbm {1}_{\{W_{t_k}\ge W_{t_{k-1}}\}} ,\quad t_{k-1}\le t\le t_{k},\quad k\in {1,\ldots ,n} , \end{aligned}$$

$G_k=\mathbbm {1}_{\{W_{t_k}\ge W_{t_{k-1}}\}}$ and the enlargement $\mathbb {G}=\mathbb {F}\vee \sigma (G_1,\ldots ,G_n)$.

## Conclusion

In this paper we show how to incorporate anticipative information in a filtration generated by a Brownian motion and a Poisson process. We compute the compensators in a general framework of additional information (see Lemma 4.2), and then we focus on the purely atomic case to consider more explicit examples (see Corollary 4.3). In particular, we study the case in which a $\mathbb {G}$-agent knows if the final pair of random variables $(W_T,N_T)$ are within a certain rectangular region, as well as the case that considers a similar type of information about the pair of running maximums $(M_T,J_T)$, see Examples 4.9 and 4.10. We also study the case when $\lambda$ is not $\mathbb {F}$-adapted in Sect. 5 and give an example when it is an anticipative function of *W*, see Example 5.3.

When the dynamics of the risky asset are driven by the Poisson process only, we give the exact value of the additional information in terms of an entropy similar to the corresponding continuous case, see Theorem 3.11. Finally, we extend that formula for the mixed case in Theorem 4.6 and Corollary 4.7.

## Data Availability

Not applicable.

## Appendix A

### Proof of Proposition 3.9

We proceed by maximizing point-wise the expression given by (31). After defining the integrand

$$\begin{aligned} I(\pi ):= \rho _s+\pi (\mu _s-\rho _s) + \lambda _s(1+\gamma _s^G)\ln (1+\pi \theta _s)-\lambda _s\pi \theta _s \end{aligned}$$

we look for each $\omega \in \Omega$, the strategy $\pi ^G_s$ that solves for each time $s\in [0,T]$ the equation $0=I'(\pi ^G_s)$. Therefore

$$\begin{aligned} 0 = \mu _s-\rho _s +\frac{\lambda _s(1+\gamma _s^G)\theta _s}{1+\pi ^G_s\theta _s} -\lambda _s\theta _s \end{aligned}$$

and solving for $\pi _s^G$ the result holds true since $I''(\pi ^G_s)<0$. $\square$

### Proof of Theorem 3.11

By (31) we have

53 $$\begin{aligned} \mathbb {V}_T^\mathbb {G}= \int _0^T \varvec{E}\left[ \rho _s + \pi _s^G(\mu _s-\rho _s) + \lambda _s(1+\gamma _s^G)\ln (1+\pi _s^G\theta _s) -\lambda _s\pi _s^G\theta _s\right] ds , \end{aligned}$$

being $\pi ^G$ the process defined in the Proposition 3.9. We compute the following terms

$$\begin{aligned} \pi _s^G\left( \mu _s-\rho _s-\lambda _s\theta _s\right)&= -\lambda _s\gamma _s^G - \frac{\mu _s-\rho _s}{\theta _s}\\ \ln \left( 1+\pi _s^G\theta _s\right)&= \ln \left( \frac{\lambda _s(1+\gamma _s^G)}{\lambda _s-(\mu _s-\rho _s)/\theta _s} \right) . \end{aligned}$$

By substituting them in (53) we have the following expression,

$$\begin{aligned} \mathbb {V}_T^\mathbb {G}&= \int _0^T \varvec{E}\left[ \rho _s - \lambda _s\gamma _s^G - \frac{\mu _s-\rho _s}{\theta _s} +\lambda _s(1+\gamma _s^G) \ln \left( \frac{\lambda _s(1+\gamma _s^G)}{\lambda _s-(\mu _s-\rho _s)/\theta _s} \right) \right] ds \end{aligned}$$

Finally, we compute the difference as follows

$$\begin{aligned} \mathbb {V}_T^\mathbb {G}-\mathbb {V}_T^\mathbb {F}&=\int _0^T \varvec{E}\left[ - \lambda _s\gamma _s^G +\lambda _s(1+\gamma _s^G) \ln \left( \frac{\lambda _s(1+\gamma _s^G)}{\lambda _s-(\mu _s-\rho _s)/\theta _s} \right) \right. \\&\quad \left. -\lambda _s \ln \left( \frac{\lambda _s}{\lambda _s-(\mu _s-\rho _s)/\theta _s} \right) \right] ds\\&= \int _0^T \varvec{E}\left[ - \lambda _s\gamma _s^G +\lambda _s(1+\gamma _s^G) \ln \left( 1+\gamma _s^G \right) \right] ds \ge 0 \end{aligned}$$

where we have applied (30) in order to simplify the expression as follows

$$\begin{aligned} \int _0^T\varvec{E}\left[ \gamma _s^G\ln \left( \frac{\lambda _s}{\lambda _s-(\mu _s-\rho _s)/\theta _s} \right) \right] ds = 0 . \end{aligned}$$

It can be checked that the function $h(x,y)=-xy+x(1+y)\ln (1+y)$ is positive when $x>0$ and $y>-1$ and we get the non-negativity of the additional gains. Finally by adding and subtracting the term $\lambda _s(1+\gamma _s^G)\ln \lambda _s$ and by applying (30) to the term $-\lambda _s\gamma _s^G$ and $\lambda _s\gamma _s^G\ln \lambda _s$ we get the result. $\square$

### Proof of Proposition 4.4

We proceed by maximizing point-wise. We define

$$\begin{aligned} I(\pi ) := \rho _s + \pi d^G_s - \frac{1}{2}\pi ^2\sigma ^2_s +c^G_s\ln (1+\pi \theta _s) \end{aligned}$$

and we consider the first order condition $0 = I'(\pi )$ as follows,

54 $$\begin{aligned} 0 = d^G_s - \pi \sigma _s^2 + c^G_s \frac{\theta _s}{1+\pi \theta _s} . \end{aligned}$$

Then we derive the next equation,

$$\begin{aligned} 0 = \left( \sigma _s^2\theta _s\right) \pi ^2 + \left( \sigma _s^2-\theta _sd^G_s\right) \pi - \left( d^G_s+\theta _sc^G_s\right) \end{aligned}$$

with the following solutions,

$$\begin{aligned} \pi ^\pm&= \frac{\theta _s d^G_s - \sigma _s^2\pm \sqrt{\left( \theta _s d^G_s-\sigma _s^2 \right) ^2 + 4\sigma _s^2\theta _s\left( d^G_s+\theta _sc^G_s\right) }}{2\sigma _s^2\theta _s}\\&= \frac{1}{2}\left( \frac{d^G_s}{\sigma _s^2}-\frac{1}{\theta _s} \right) \pm \frac{{{\,\textrm{sgn}\,}}(\theta _s)}{2} \sqrt{\left( \frac{d^G_s}{\sigma _s^2}+\frac{1}{\theta _s}\right) ^2+4 \frac{c^G_s}{\sigma _s^2}} , \end{aligned}$$

where in the last step we have used arithmetic computations. It can be verified that $I''(0)<0$ and the pair of strategies $\pi ^\pm$ are maximum if and only if they are admissible. Then we need to check if the condition $1+\pi ^{\pm }\theta _s > 0$ is satisfied. We rewrite the pair as follows,

55 $$\begin{aligned} 1 + \pi ^{\pm }\theta _s = \frac{1}{2}\left( \frac{d^G_s\theta _s}{\sigma _s^2} + 1 \right) \pm \frac{1}{2} \sqrt{\left( \frac{d^G_s\theta _s}{\sigma _s^2} + 1 \right) ^2 + 4\theta _s^2\frac{c^G_s}{\sigma _s^2}} \end{aligned}$$

and we deduce that the unique optimal solution is $\pi ^+=(\pi ^+_t, \, 0 \le {t} \le {T})$,

then the result holds with the fact that $I''(\pi ^+)<0$. $\square$

### Proof of Theorem 4.6

We recall the notation of $d^G_s$ and $c^G_s$ in the previous computation. Using (54),

$$\begin{aligned} \mathbb {V}_T^{\mathbb {G}}&= \int _0^{T}\varvec{E}\Big [ \rho _s + \pi _s d^G_s - \frac{\pi ^2_s\sigma ^2_s}{2} + c^G_s \ln (1+\pi _s\theta _s)\Big ]ds\\&= \int _0^{T}\varvec{E}\Big [ \rho _s + \frac{\pi ^2_s\sigma ^2_s}{2} -c^G_s \left( \frac{\pi _s\theta _s}{1+\pi _s\theta _s} + \ln \left( \frac{1}{1+\pi _s\theta _s}\right) \right) \Big ]ds\\&= \int _0^{T}\varvec{E}\Big [ \rho _s + \frac{\pi ^2_s\sigma ^2_s}{2} -c^G_s +c^G_s \left( \frac{1}{1+\pi _s\theta _s} - \ln \left( \frac{1}{1+\pi _s\theta _s}\right) \right) \Big ]ds \end{aligned}$$

Note that, using (55)

$$\begin{aligned} (\pi _s\sigma _s)^2&= \frac{1}{2}\left( \frac{d^G_s}{\sigma _s}\right) ^2 +\frac{1}{2} \left( \frac{\sigma _s}{\theta _s}\right) ^2 +c^G_s\\&\quad + \frac{1}{2}\left( \frac{d^G_s}{\sigma _s}-\frac{\sigma _s}{\theta _s}\right) {{\,\textrm{sgn}\,}}(\theta _s)\sqrt{\left( \frac{d^G_s}{\sigma _s}+\frac{\sigma _s}{\theta _s}\right) ^2+4c^G_s}\\ \frac{c^G_s}{1+\pi _s\theta _s}&= \frac{c^G_s}{2}\frac{ \sqrt{\left( \frac{d^G_s\theta _s}{\sigma _s^2} + 1 \right) ^2 + 4\frac{\theta _s^2}{\sigma _s^2}c^G_s} - \left( \frac{d^G_s\theta _s}{\sigma _s^2} + 1 \right) }{\frac{\theta _s^2}{\sigma _s^2}c^G_s} \\&=\frac{\sigma _s^2}{2\theta _s^2}\left( \sqrt{\left( \frac{d^G_s\theta _s}{\sigma _s^2} + 1 \right) ^2 + 4\frac{\theta _s^2}{\sigma _s^2}c^G_s} - \left( \frac{d^G_s\theta _s}{\sigma _s^2} + 1 \right) \right) \\&=\frac{\sigma _s}{2\theta _s}\left( {{\,\textrm{sgn}\,}}(\theta _s)\sqrt{\left( \frac{d^G_s}{\sigma _s}+\frac{\sigma _s}{\theta _s} \right) ^2 + 4c^G_s} - \left( \frac{d^G_s}{\sigma _s} + \frac{\sigma _s}{\theta _s} \right) \right) \\ -c^G_s \ln \left( \frac{1}{1+\pi _s\theta _s}\right)&= c^G_s\ln c^G_s + c^G_s \ln \left( 2\frac{\theta _s^2}{\sigma _s^2}\right) \\&\quad - c^G_s\ln \left( \sqrt{\left( \frac{d^G_s\theta _s}{\sigma _s^2} + 1 \right) ^2 + 4\frac{\theta _s^2}{\sigma _s^2}c^G_s} - \left( \frac{d^G_s\theta _s}{\sigma _s^2} + 1 \right) \right) \end{aligned}$$

By rearranging all the terms we get

$$\begin{aligned} \mathbb {V}_T^\mathbb {G}&= \int _0^T\varvec{E}\Bigg [ \frac{1}{2} \left( \frac{d^G_s}{\sigma _s}\right) ^2+ \frac{1}{2}\frac{d^G_s}{\sigma _s}{{\,\textrm{sgn}\,}}(\theta _s)\sqrt{\left( \frac{d^G_s}{\sigma _s}+\frac{\sigma _s}{\theta _s}\right) ^2+4c^G_s} -\frac{d^G_s}{2\theta _s} +c^G_s\ln c^G_s\\&\quad +c^G_s \ln \left( 2\frac{\theta _s^2}{\sigma _s^2}\right) - c^G_s\ln \left( \sqrt{\left( \frac{d^G_s\theta _s}{\sigma _s^2} + 1 \right) ^2 + 4\frac{\theta _s^2}{\sigma _s^2}c^G_s} - \left( \frac{d^G_s\theta _s}{\sigma _s^2} + 1 \right) \right) \Bigg ] ds , \end{aligned}$$

and by simplifying the terms $-\dfrac{d^G_s}{2\theta _s}$ and $c^G_s \ln \left( 2\dfrac{\theta _s^2}{\sigma _s^2}\right)$ because of (30), the result follows true. $\square$

### Proof of Proposition 5.2

Let $A\in \mathcal {F}_s$ and $B\in \sigma (G)$, then by (35) we have

$$\begin{aligned}&\varvec{E}[\mathbbm {1}_A\mathbbm {1}_{\{G\in B\}}({\tilde{N}}_t-{\tilde{N}}_s)] \\&\quad = \varvec{E}\left[ \mathbbm {1}_A\left( \varvec{P}^G(B\vert \mathcal {F}_T^\lambda )+ \int _0^T\int _B \zeta ^g_s \varvec{P}^G(dg) dW_s + \int _0^T \int _B \psi ^{g}_s \varvec{P}^G(dg) d{\tilde{N}}_s\right) ({\tilde{N}}_t-{\tilde{N}}_s)\right] . \end{aligned}$$

For the first term we have

$$\begin{aligned} \varvec{E}\left[ \mathbbm {1}_A \varvec{P}^G(B\vert \mathcal {F}_T^\lambda )({\tilde{N}}_t-\tilde{N}_s)\right]&= \varvec{E}\left[ \mathbbm {1}_A \varvec{P}^G(B\vert \mathcal {F}_T^\lambda )\varvec{E}[({\tilde{N}}_t-{\tilde{N}}_s)\vert \mathcal {F}_s \vee \mathcal {F}_T^\lambda ]\right] \\&= \varvec{E}\left[ \mathbbm {1}_A \varvec{P}^G(B\vert \mathcal {F}_T^\lambda )\varvec{E}[\int _s^t{\bar{\gamma }}_u^\lambda \,du\vert \mathcal {F}_s \vee \mathcal {F}_T^\lambda ]\right] \\&= \varvec{E}\left[ \mathbbm {1}_A \varvec{P}^G(B\vert \mathcal {F}_T^\lambda )\int _s^t{\bar{\gamma }}_u^\lambda \,du\right] \\&= \varvec{E}\left[ \mathbbm {1}_A \int _s^t\int _B{\bar{\gamma }}_u^\lambda \varvec{P}^G(dg\vert \mathcal {F}_T^\lambda )\,du\right] \\&=\varvec{E}\left[ \mathbbm {1}_A \int _s^t\int _B{{\widetilde{\gamma }}}_u^\lambda \varvec{P}^G(dg\vert \mathcal {F}_u\vee \mathcal {F}_T^\lambda )\,du\right] \\&=\varvec{E}\left[ \mathbbm {1}_A \int _s^t\varvec{E}[\mathbbm {1}_{\{G\in B\}} {{\widetilde{\gamma }}}^\lambda _u \vert \mathcal {F}_u\vee \mathcal {F}_T^\lambda ]\,du\right] \\&=\varvec{E}\left[ \mathbbm {1}_A \mathbbm {1}_{\{G\in B\}}\int _s^t {{\widetilde{\gamma }}}^\lambda _u \,du\right] . \end{aligned}$$

With respect to the second term we use the fact that ${\widehat{W}}_\cdot :=W_\cdot -\int _0^\cdot {\widehat{\alpha }}^G_uds$ is a $\mathbb {F}\vee \mathbb {F}^\lambda$-martingale, then

$$\begin{aligned}&\varvec{E}\left[ \mathbbm {1}_A \int _0^T\int _B \zeta ^g_s \varvec{P}^G(dg) dW_s({\tilde{N}}_t-{\tilde{N}}_s)\right] \\&\quad =\varvec{E}\left[ \mathbbm {1}_A \left( \int _0^T\int _B \zeta ^g_u \varvec{P}^G(dg) d{{\widehat{W}}}_u + \int _0^T\int _B \zeta ^g_u \varvec{P}^G(dg) \widehat{\alpha }^\lambda _udu \right) ({\tilde{N}}_t-{\tilde{N}}_s)\right] \\&\quad =\varvec{E}\left[ \mathbbm {1}_A \int _0^T\int _B \zeta ^g_u \varvec{P}^G(dg) \widehat{\alpha }^\lambda _udu\, ({\tilde{N}}_t-{\tilde{N}}_s)\right] , \end{aligned}$$

where in the second equality we applied the Itô isometry for $\mathbb {F}\vee \mathbb {F}^\lambda$-martingales. The latter integral in [0, *T*] is going to be split in [0, *s*], [*s*, *t*] and [*t*, *T*]. To short the notation we introduce the $\mathbb {F}$-adapted process $Y^B_u:= \int _B \zeta ^g_u \varvec{P}^G(dg)$, $u\in [0,T]$. For the first interval we have,

$$\begin{aligned} \varvec{E}\left[ \mathbbm {1}_A\int _0^sY^B_u {\widehat{\alpha }}^\lambda _udu\, (\tilde{N}_t-{\tilde{N}}_s)\right]&= \varvec{E}\left[ \int _s^t \mathbbm {1}_A \int _0^sY^B_u {\widehat{\alpha }}^\lambda _udu\,d{\tilde{N}}_v\right] = 0 , \end{aligned}$$

where we used that ${\tilde{N}}$ is a $\mathbb {F}\vee \mathbb {F}^\lambda$ martingale and it is the expectation of a well-define Itô integral. For the interval [*t*, *T*],

56 $$\begin{aligned} \varvec{E}\left[ \mathbbm {1}_A\int _t^TY^B_u {\widehat{\alpha }}^\lambda _udu\,(\tilde{N}_t-{\tilde{N}}_s)\right]&=-\varvec{E}\left[ \mathbbm {1}_A\int _s^t\int _t^T\lambda _vY_u^B{\widehat{\alpha }}^\lambda _u\,dudv\right] \nonumber \\&=-\varvec{E}\left[ \mathbbm {1}_A\int _s^t\left( \int _v^T-\int _v^t\right) \lambda _vY_u^B\widehat{\alpha }^\lambda _u\,dudv\right] \end{aligned}$$

and we continue with the first integral.

$$\begin{aligned}&\varvec{E}\left[ \mathbbm {1}_A\int _s^t\int _v^T\lambda _vY^B_u\widehat{\alpha }^\lambda _u\,dudv\right] = \varvec{E}\left[ \mathbbm {1}_A\int _s^t\int _v^T\lambda _v\int _B {\widehat{\gamma }}^g_u\varvec{P}^G(dg\vert \mathcal {F}_u,\mathcal {F}_u^\lambda )\,dudv\right] \\&\quad = \varvec{E}\left[ \mathbbm {1}_A\int _s^t\int _v^T\lambda _v\varvec{E}[\mathbbm {1}_{\{G\in B\}}{\widehat{\gamma }}_u^G\vert \mathcal {F}_u,\mathcal {F}_u^\lambda ]\,dudv\right] = \varvec{E}\left[ \mathbbm {1}_A\mathbbm {1}_{\{G\in B\}}\int _s^t\int _v^T\lambda _v{\widehat{\gamma }}_u^G\,dudv\right] . \end{aligned}$$

Next we prove that the interval [*s*, *t*] simplifies with the second integral in (56). Indeed,

57 $$\begin{aligned}&\varvec{E}\left[ \mathbbm {1}_A \int _s^tY^B_u {\widehat{\alpha }}^\lambda _udu\, ({\tilde{N}}_t-{\tilde{N}}_s)\right] = \varvec{E}\left[ \mathbbm {1}_A \int _s^t \left( \int _s^v Y^B_u {\widehat{\alpha }}^\lambda _udu+ \int _v^tY^B_u {\widehat{\alpha }}^\lambda _udu\right) d{\tilde{N}}_v\right] \nonumber \\&\quad = \varvec{E}\left[ \mathbbm {1}_A \int _s^t \int _v^tY^B_u \widehat{\alpha }^\lambda _udud{\tilde{N}}_v\right] =\varvec{E}\left[ \mathbbm {1}_A \int _s^t ({\tilde{N}}_u-{\tilde{N}}_s)Y^B_u {\widehat{\alpha }}^\lambda _u\,du\right] \nonumber \\&\quad =-\varvec{E}\left[ \mathbbm {1}_A \int _s^t \int _s^u\lambda _vdvY^B_u \widehat{\alpha }^\lambda _u\,du\right] = -\varvec{E}\left[ \mathbbm {1}_A \int _s^t \int _v^t\lambda _vY^B_u {\widehat{\alpha }}^\lambda _u\,dudv\right] , \end{aligned}$$

where we used that $N_u-N_s$, $s\le u$, is $\mathbb {F}$-adapted and for any $\mathbb {F}$-adapted process $X=(X_t, \, 0 \le {t} \le {T})$ we have

$$\begin{aligned} 0 = \varvec{E}\left[ \int _0^T X_u\,d{{\widehat{W}}}_u-\int _0^T X_u\,d W_u\right] = \varvec{E}\left[ \int _0^T X_u{\widehat{\alpha }}^\lambda _u\,du\right] . \end{aligned}$$

We consider now

$$\begin{aligned}&\varvec{E}\left[ \mathbbm {1}_A\int _0^T \int _B \psi ^{g}_s \varvec{P}^G(dg) d\tilde{N}_s({\tilde{N}}_t-{\tilde{N}}_s)\right] = \varvec{E}\left[ \mathbbm {1}_A \int _s^t\int _B \psi ^g_u \varvec{P}^G(dg) dN_u \right] \\&\quad = \varvec{E}\left[ \mathbbm {1}_A \int _s^t\int _B \lambda _u\gamma _u^g\varvec{P}^G(dg\vert \mathcal {F}_u) du \right] = \varvec{E}\left[ \mathbbm {1}_A\mathbbm {1}_{\{G\in B\}} \int _s^t\lambda _u\gamma _u^Gdu \right] , \end{aligned}$$

where in the first equality we used the Itô isometry and the fact that $[{\tilde{N}}_\cdot ]^{\mathbb {F}\vee \mathbb {F}^\lambda } = N_\cdot$, in the second one we applied the definition of $\gamma ^G$ while in the third one we used the properties of the conditional expectation operator. By putting together all the terms and taking $\mathcal {G}_t$-conditional expectation, we conclude that

$$\begin{aligned} N_\cdot - \int _0^\cdot \left( \lambda _v+{{\widetilde{\gamma }}}_v^\lambda + \lambda _v\gamma ^G_v -\lambda _v \varvec{E}\left[ \int _v^T{\widehat{\gamma }}^G_udu\vert \mathcal {G}_v\right] \right) dv \end{aligned}$$

is a $\mathbb {G}$-local martingale. To finish the proof, we sketch the computation in order to calculate the information drift of *W*.

$$\begin{aligned}&\varvec{E}[\mathbbm {1}_A\mathbbm {1}_{\{G\in B\}}(W_t-W_s)] \\&\quad = \varvec{E}\left[ \mathbbm {1}_A\left( \varvec{P}^G(B\vert \mathcal {F}_T^\lambda )+ \int _0^T\int _B \zeta ^g_s \varvec{P}^G(dg) dW_s + \int _0^T \int _B \psi ^{g}_s \varvec{P}^G(dg) d{\tilde{N}}_s\right) (W_t-W_s)\right] . \end{aligned}$$

For the case of the two first terms we have

$$\begin{aligned} \varvec{E}\left[ \mathbbm {1}_A\varvec{P}^G(B\vert \mathcal {F}_T^\lambda )(W_t-W_s)\right]&= \varvec{E}\left[ \mathbbm {1}_A\varvec{P}^G(B\vert \mathcal {F}_T^\lambda )\int _s^t{\bar{\alpha }}^\lambda _udu\right] \\&= \varvec{E}\left[ \mathbbm {1}_A\mathbbm {1}_{\{G\in B\}}\int _s^t{{\widetilde{\alpha }}}^\lambda _udu\right] ,\\ \varvec{E}\left[ \mathbbm {1}_A\int _0^T\int _B \zeta ^g_s \varvec{P}^G(dg) dW_s (W_t-W_s)\right]&= \varvec{E}\left[ \mathbbm {1}_A\mathbbm {1}_{\{G\in B\}} \int _s^t\alpha _u^Gdu \right] . \end{aligned}$$

By defining $Z_u^B:= \int _B \psi ^{g}_s \varvec{P}^G(dg)$, we proceed entirely as in the Poisson case. We omit some details that can be consulted in the first part of the proof.

$$\begin{aligned}&\varvec{E}\left[ \mathbbm {1}_A\int _0^T Z_v^B d{\tilde{N}}_v(W_t-W_s)\right] =\varvec{E}\left[ \mathbbm {1}_A\int _0^T Z_v^B d{\tilde{N}}_v\left( {{\widehat{W}}}_t-{{\widehat{W}}}_s+\int _s^t{\widehat{\alpha }}_u^\lambda du\right) \right] \\&\quad =\varvec{E}\left[ \mathbbm {1}_A\int _0^T Z_v^B d\tilde{N}_v\int _s^t{\widehat{\alpha }}_u^\lambda du\right] = \varvec{E}\left[ \mathbbm {1}_A\left( \int _0^s+\int _s^t\right) Z_v^B d\tilde{N}_v\int _s^t{\widehat{\alpha }}_u^\lambda du\right] . \end{aligned}$$

For the first integral we have

$$\begin{aligned} \varvec{E}\left[ \mathbbm {1}_A\int _0^s Z_v^B d\tilde{N}_v\int _s^t{\widehat{\alpha }}_u^\lambda du\right] = -\varvec{E}\left[ \mathbbm {1}_A\int _s^t\left( \int _0^u-\int _s^u \right) Z_v^B \lambda _v{\widehat{\alpha }}_u^\lambda \,dv du\right] . \end{aligned}$$

As in the Poisson case, the second integral is simplified with the [*s*, *t*] interval. So we deal with the first one,

$$\begin{aligned} \varvec{E}\left[ \mathbbm {1}_A\int _s^t\int _0^u Z_v^B \lambda _v{\widehat{\alpha }}_u^\lambda \,dv du\right]&= \varvec{E}\left[ \mathbbm {1}_A\int _s^t\int _0^u \varvec{E}[\mathbbm {1}_{\{G\in B\}}\lambda _v\gamma ^G_v\vert \mathcal {F}_v]{\widehat{\alpha }}_u^\lambda \,dv du\right] = 0. \end{aligned}$$

So we conclude that the process

$$\begin{aligned} W_\cdot - \int _0^\cdot \left( {{\widetilde{\alpha }}}^\lambda _v+\alpha ^G_v\right) dv \end{aligned}$$

is a $\mathbb {G}$-martingale and the result holds true. $\square$
